# Supplementary material for: Critical review of healthcare financing and a survey of system quality perception among healthcare users in Nigeria (2010–2023)
Source: PLOS Glob Public Health. 2025 May 28;5(5):e0004615. doi: 10.1371/journal.pgph.0004615 (PMC12118842; doi:10.1371/journal.pgph.0004615)
Supplement: S1 Checklist — is a PLOS policy on inclusivity in global research, which focuses on ensuring that research conducted outside of a researcher’s home country is reported transparently and ethically. (DOCX) [file pgph.0004615.s001.docx]

Inclusivity in global research

PLOS’ policy on inclusivity in global research aims to improve transparency in the reporting of research performed outside of researchers’ own country or community and ensures that PLOS publications reporting global research adhere to high standards for research ethics and authorship. Authors of relevant research articles may be asked to complete the questionnaire below, which outlines ethical, cultural, and scientific considerations specific to inclusivity in global research. This questionnaire may be requested when researchers have travelled to a different country to conduct research, if research uses samples collected in another country, research with Indigenous populations or their lands, or if research is on cultural artefacts. Researchers travelling to another country solely to use laboratory equipment will not normally be required to complete the questionnaire. However, the questionnaire can be requested at the journal’s discretion for any submission – if you have been requested to complete this questionnaire by the PLOS journal you submitted to, please do so.

Please complete the questionnaire below and include this as a Supporting Information file with your manuscript. Note that if your paper is accepted for publication, this checklist will be published with your article in the supporting information files. Please ensure that you reference the checklist in the main body of your manuscript. We suggest adding a subsection ‘Inclusivity in global research’ to your Methods section and adding the following sentence: “Additional information regarding the ethical, cultural, and scientific considerations specific to inclusivity in global research is included in the Supporting Information (SX Checklist)”

The questions have been designed to be applicable to a wide range of study types, and there are subsections for both human subjects research and non-human subjects research. If any of the questions are not relevant to your research please mark them as “N/A” as appropriate.

**Ethical considerations, permits and authorship**

*This section is applicable to all research types.*

Provide details as to who granted permissions and/or consent for the study to take place in the Methods section of your manuscript. This should include the names of **all** ethics boards, governmental organizations, community leaders or other bodies that provided approval for the study. If individuals provided approval refer to these people by their role or title but do not list their name(s).

Reported on page number: 7-8. The research was conducted in public spaces within reasonable catchment areas of randomly identified health facilities. The ethical approval letter gotten from the National Health Research Ethics Committee was honored by respondents. Where necessary, the managements of public organizations were approached with same approval document for verbal permission to conduct the research. There was no direct or indirect resistance to data collection and the research was warmly embraced. The ethical clearance from the authority is attached to the submission.

If there were any deviations from the study protocol after approval was obtained please provide details of these changes in the Methods section of your manuscript.

No significant deviation on the protocol, however, as reported on page number: 8

There are no significant changes to the protocol other than the separate survey between healthcare workers (manuscript 1 already published, see <https://doi.org/10.1371/journal.pgph.0003881>) and the general population (this manuscript 2). Each manuscript required two suitable titles although the original protocol was approval was given as “*Critical review of healthcare financing and end-users’ quality perception in Nigeria*”.

Did this study involve local collaborators that are residents of the country where the research was conducted or members of the community studied? If you do not have any authors from said communities, please provide an explanation for this below.

Yes.

Prisca Olabisi Adejumo, Mercy Emmanue, Oyinye Prosper Martins-Ifeanyi, Ufuomaoghene Jemima Mukoro, Fawole Israel Opeyemi, Timothy Wale Olasebikan, Abosede Peace Adebayo, Deborah Oyinlola Salawu, Busiroh Mobolape Ibraheem, Ubiebo Ataisi Ekenekot, Precious Ebinehita Imoyera, Mudiaga Sidney Edafiejire, Joy Chioma Obialor, Gloria Oluwakorede Alao, Blessing Onyinye Obialor, Olukayode Joseph Oladimeji, and Ajao Adewale Gbolabo are co-authors who were based full-time in Nigeria during this research.

Everyone listed as an author should meet PLOS’ criteria for authorship and all individuals who meet these criteria should be included in the author byline, rather than the acknowledgements. For further information please see the journal’s Authorship Policy.

**Human subjects research (e.g. health research, medical research, cross-cultural psychology)**

Did you obtain written informed consent from a representative of the local community or region before the research took place? How did you establish who speaks for the community? Details of written informed consent obtained from study participants should be reported separately in the Methods section of your manuscript.

Page 10 has the details on ethical clearance, permissions, and informed consent information

How did members of the local community provide input on the aims of the research investigation, its methodology, and its anticipated outcome(s)?

All respondents were able to read the informed consent documents written in English language which is the official language in Nigeria.

When engaging with the local community, how did you ensure that the informed consent documents and other materials could be understood by local stakeholders?

We used verbal explanations only where necessary during the survey. This was used to augment print or digital informed consent forms as well as a detailed informed consent on the introduction part of each questionnaire requiring a yes or no agreement to continue with the study.

Will the findings of the research be made available in an understandable format to stakeholders in the community where the study was conducted (e.g. via a presentation, summary report, copies of publications, etc.)? Please provide details of how this will be achieved.

Copies of publications will be shared with the Nigerian Health Research Ethics Committee (NREC) and all the facilities from which data were collected. Submitting the publication report to the NREC is a requirement in the approval process that we are adhering to. The first publication (<https://doi.org/10.1371/journal.pgph.0003881>) has been shared with the NREC.

**Non-human subjects research using specimens/ animals collected as part of the study, or those housed in archival collections. Examples include archaeology, paleontology, botany and zoology.**

Did the permission you obtained from a local authority to perform the study include an agreement on access to outputs and benefit sharing? This may include procedures to enable fair distribution of the benefits and resources arising from the research performed. Please include any details of Prior Informed Consent and Benefit Sharing Agreements obtained. These may be required by field-specific regulations, for example the Convention on Biological Diversity (CBD) and the associated Nagoya Protocol.

Not applicable

If the material used in your study was imported, please A) provide the year it was imported and B) indicate whether permits were obtained to import/export the materials used, C) provide details of any permits obtained. If this information is not available, please indicate this.

All budget data were publicly available (with open access) on the official government repository and was accessed with the guidance of Ministry of Budget and Economic Planning Abuja, Nigeria.

If you used archival specimens, please state how the material used in your study was acquired by the institute it is held in and provide details of any permits obtained for the original excavations/ sample collection. If this information is not available, please indicate this.

Not applicable

How was the potential cultural significance of the materials collected in your study to local communities considered in your research design? Were Indigenous peoples and/or local researchers and institutions involved with archaeological excavations / collection of specimens? If so, please provide a description of their involvement.

Not applicable

If your manuscript includes photographs of human remains please indicate whether authors obtained permission from descendants or affiliated cultural communities to do so.

Not applicable
