# Supplementary material for: Critical review of healthcare financing and a survey of system quality perception among healthcare users in Nigeria (2010–2023)
Source: PLOS Glob Public Health. 2025 May 28;5(5):e0004615. doi: 10.1371/journal.pgph.0004615 (PMC12118842; doi:10.1371/journal.pgph.0004615)
Supplement: S1 Table — provides a detailed representation of the crosstabulation of Sociodemographic attributes and average rating of the quality of the healthcare system. (DOCX) [file pgph.0004615.s002.docx]

S1 Table: Crosstabulation of Sociodemographic attributes and average rating of the quality of the healthcare system.

|  | | **Average Rating** | | | | | **Total** |
| --- | --- | --- | --- | --- | --- | --- | --- |
|  |  | **1** | **2** | **3** | **4** | **5** |  |
| Gender | Male | 170 | 585 | 358 | 157 | 10 | 1280 |
|  | Female | 109 | 483 | 204 | 126 | 10 | 932 |
| Total | | 279 | 1068 | 562 | 283 | 20 | 2212 |
| Age | 30-39 years | 131 | 409 | 202 | 118 | 7 | 867 |
|  | 40-49 years | 102 | 402 | 221 | 77 | 12 | 814 |
|  | 50-59 years | 40 | 198 | 96 | 61 | 1 | 396 |
|  | 60 and above | 6 | 59 | 43 | 27 | 0 | 135 |
| Total | | 279 | 1068 | 562 | 283 | 20 | 2212 |
| Select the one that best or most closely describe your State | Delta | 17 | 72 | 50 | 30 | 0 | 169 |
|  | Enugu | 42 | 87 | 4 | 2 | 0 | 135 |
|  | Kaduna | 7 | 108 | 63 | 70 | 12 | 260 |
|  | Kano | 100 | 215 | 52 | 9 | 1 | 377 |
|  | Kwara | 58 | 73 | 14 | 13 | 0 | 158 |
|  | Lagos | 15 | 233 | 151 | 55 | 3 | 457 |
|  | Oyo | 11 | 101 | 55 | 69 | 3 | 239 |
|  | Plateau | 10 | 67 | 30 | 7 | 0 | 114 |
|  | Rivers | 19 | 112 | 143 | 28 | 1 | 303 |
| Total | | 279 | 1068 | 562 | 283 | 20 | 2212 |
| Education | High school or below | 54 | 185 | 88 | 32 | 1 | 360 |
|  | Diploma | 77 | 205 | 70 | 60 | 5 | 417 |
|  | Bachelor | 104 | 458 | 270 | 109 | 10 | 951 |
|  | Master/Postgraduate | 38 | 188 | 106 | 66 | 3 | 401 |
|  | PhD/Fellowships | 6 | 32 | 28 | 16 | 1 | 83 |
| Total | | 279 | 1068 | 562 | 283 | 20 | 2212 |
| Employment | Full-time Employment | 180 | 724 | 373 | 176 | 8 | 1461 |
|  | Part-time employment | 58 | 185 | 78 | 61 | 9 | 391 |
|  | Unemployed | 41 | 159 | 111 | 46 | 3 | 360 |
| Total | | 279 | 1068 | 562 | 283 | 20 | 2212 |
| Employer | Government | 96 | 416 | 186 | 123 | 3 | 824 |
|  | Private/Not Government | 87 | 291 | 143 | 53 | 8 | 582 |
|  | Self-employed | 96 | 361 | 233 | 107 | 9 | 806 |
| Total | | 279 | 1068 | 562 | 283 | 20 | 2212 |
| Which category best describes how much you earn per month in Naira? | Less than 35,000 | 70 | 158 | 93 | 36 | 1 | 358 |
|  | 35,000 to 49,000 | 67 | 195 | 54 | 34 | 2 | 352 |
|  | 50,000 to 99,000 | 70 | 290 | 164 | 60 | 1 | 585 |
|  | 100,000 to 199,000 | 42 | 206 | 127 | 72 | 6 | 453 |
|  | 200,000 to 399,000 | 20 | 170 | 81 | 43 | 7 | 321 |
|  | More than 400,000 | 10 | 49 | 43 | 38 | 3 | 143 |
| Total | | 279 | 1068 | 562 | 283 | 20 | 2212 |
| Field of Employment [Select the group that best describes your job] | Agriculture and Lands | 10 | 44 | 23 | 14 | 0 | 91 |
|  | Banking, Trade, Marketing, and Financial Services | 32 | 91 | 66 | 29 | 1 | 219 |
|  | Cultural, Legal, and Social Services | 21 | 96 | 84 | 19 | 2 | 222 |
|  | Education | 26 | 122 | 77 | 25 | 5 | 255 |
|  | Engineering, Mining, and Architecture | 26 | 88 | 47 | 17 | 3 | 181 |
|  | Entertainment and Arts | 19 | 86 | 48 | 16 | 2 | 171 |
|  | Government, Politics, and Administration | 28 | 77 | 41 | 12 | 2 | 160 |
|  | Healthcare | 43 | 163 | 50 | 65 | 4 | 325 |
|  | I earn just enough to for me and my dependents | 0 | 1 | 0 | 0 | 0 | 1 |
|  | Information, Technology, and Communication Services | 22 | 93 | 42 | 31 | 0 | 188 |
|  | Student | 34 | 106 | 40 | 26 | 1 | 207 |
|  | Transportation and logistics | 0 | 3 | 5 | 3 | 0 | 11 |
|  | Unemployed | 18 | 98 | 39 | 26 | 0 | 181 |
| Total | | 279 | 1068 | 562 | 283 | 20 | 2212 |
