# Supplementary material for: Critical review of healthcare financing and a survey of system quality perception among healthcare users in Nigeria (2010–2023)
Source: PLOS Glob Public Health. 2025 May 28;5(5):e0004615. doi: 10.1371/journal.pgph.0004615 (PMC12118842; doi:10.1371/journal.pgph.0004615)
Supplement: S2 Table — provides a detailed representation of the crosstabulation of sociodemographic attributes and preference for healthcare facilities. (DOCX) [file pgph.0004615.s003.docx]

S2 Table: Crosstabulation of Sociodemographic attributes and preference of healthcare facilities.

|  | | Do you prefer government or private healthcare facilities? | | | Total |
| --- | --- | --- | --- | --- | --- |
|  |  | Government | Private | No Preference |  |
| Gender | Male | 895 | 154 | 231 | 1280 |
|  | Female | 685 | 101 | 146 | 932 |
| Total | | 1580 | 255 | 377 | 2212 |
| Age | 30-39 years | 672 | 85 | 110 | 867 |
|  | 40-49 years | 540 | 94 | 180 | 814 |
|  | 50-59 years | 265 | 63 | 68 | 396 |
|  | 60 and Above | 103 | 13 | 19 | 135 |
| Total | | 1580 | 255 | 377 | 2212 |
| Select the one that best or most closely describe your State | Delta | 163 | 2 | 4 | 169 |
|  | Enugu | 81 | 13 | 41 | 135 |
|  | Kaduna | 165 | 21 | 74 | 260 |
|  | Kano | 287 | 40 | 50 | 377 |
|  | Kwara | 70 | 69 | 19 | 158 |
|  | Lagos | 297 | 36 | 124 | 457 |
|  | Oyo | 218 | 13 | 8 | 239 |
|  | Plateau | 76 | 24 | 14 | 114 |
|  | Rivers | 223 | 37 | 43 | 303 |
| Total | | 1580 | 255 | 377 | 2212 |
| Education | High school or below | 279 | 37 | 44 | 360 |
|  | Diploma | 303 | 52 | 62 | 417 |
|  | Bachelor | 635 | 115 | 201 | 951 |
|  | Master/Postgraduate | 291 | 46 | 64 | 401 |
|  | PhD/Fellowships | 72 | 5 | 6 | 83 |
| Total | | 1580 | 255 | 377 | 2212 |
| Employment | Full-time Employment | 1013 | 169 | 279 | 1461 |
|  | Part-time employment | 275 | 56 | 60 | 391 |
|  | Unemployed | 292 | 30 | 38 | 360 |
| Total | | 1580 | 255 | 377 | 2212 |
| Employer | Government | 585 | 100 | 139 | 824 |
|  | Private/Not Government | 392 | 55 | 135 | 582 |
|  | Self-employed | 603 | 100 | 103 | 806 |
| Total | | 1580 | 255 | 377 | 2212 |
| Which category best describes how much you earn per month in Naira? | Less than 35,000 | 334 | 1 | 23 | 358 |
|  | 35,000 to 49,000 | 272 | 65 | 15 | 352 |
|  | 50,000 to 99,000 | 396 | 106 | 83 | 585 |
|  | 100,000 to 199,000 | 196 | 2 | 255 | 453 |
|  | 200,000 to 399,000 | 264 | 56 | 1 | 321 |
|  | More than 400,000 | 118 | 25 | 0 | 143 |
| Total | | 1580 | 255 | 377 | 2212 |
| Field of Employment [Select the group that best describes your job] | Agriculture and Lands | 58 | 17 | 16 | 91 |
|  | Banking, Trade, Marketing, and Financial Services | 163 | 20 | 36 | 219 |
|  | Cultural, Legal, and Social Services | 154 | 22 | 46 | 222 |
|  | Education | 197 | 29 | 29 | 255 |
|  | Engineering, Mining, and Architecture | 130 | 23 | 28 | 181 |
|  | Entertainment and Arts | 114 | 27 | 30 | 171 |
|  | Government, Politics, and Administration | 107 | 13 | 40 | 160 |
|  | Healthcare | 242 | 26 | 57 | 325 |
|  | I earn just enough to for me and my dependents | 1 | 0 | 0 | 1 |
|  | Information, Technology, and Communication Services | 139 | 27 | 22 | 188 |
|  | Student | 139 | 34 | 34 | 207 |
|  | Transportation and logistics | 8 | 2 | 1 | 11 |
|  | Unemployed | 128 | 15 | 38 | 181 |
| Total | | 1580 | 255 | 377 | 2212 |
