# Supplementary material for: Critical review of healthcare financing and a survey of system quality perception among healthcare users in Nigeria (2010–2023)
Source: PLOS Glob Public Health. 2025 May 28;5(5):e0004615. doi: 10.1371/journal.pgph.0004615 (PMC12118842; doi:10.1371/journal.pgph.0004615)
Supplement: Attachment — Submitted filename: PGPH-D-24-02908_EO.pdf [file pgph.0004615.s004.pdf]

PLOS Global Public Health

Critical Review of Healthcare Financing and System Quality Perception among Healthcare Users in Nigeria

--Manuscript Draft--

**Manuscript Number:**	PGPH-D-24-02908
**Article Type:**	Research Article
**Full Title:**	Critical Review of Healthcare Financing and System Quality Perception among Healthcare Users in Nigeria
**Short Title:**	Healthcare Financing and Quality in Nigeria
**Corresponding Author:**	Blessing O. Josiah, RN,BSN,MHM,MIPH<br>Turks and Caicos Islands Community College<br>Grand Turk, TURKS AND CAICOS ISLANDS
**Order of Authors:**	Blessing O. Josiah, RN,BSN,MHM,MIPH
	Emmanuel Chukwunwike Enebeli
	Brontie Albertha Duncan
	Lordsfavour Anukam
	Muhammad Baqir Shittu
	Oluwadamilare Akingbade
	France Ncube
	Chinelo Cleopatra Josiah
	Kelechi Eric Alimele
	Mercy Emmanuel
	Oyinye Prosper Martins-Ifeanyi
	Fawole Israel Opeyemi
	Abosede Peace Adebayo
	Deborah Oyinlola Salawu
	Busiroh Mobolape Ibraheem
	Ubiebo Ataisi Ekenekot
	Precious Ebinehita Imoyera
	Mudiaga Sidney Edafiejire
	Joy Chioma Obialor
	Gloria Oluwakorede Alao
	Blessing Onyinye Obialor
	Ajao Adewale Gbolabo
	Siyouneh Baghdasarian
	Solomon Oluwaseun Olukoya
	Ndidi Louis Otoboyor
	Oghosa Gabriel Josiah
	Ufuomaoghene Jemima Mukoro
	Blessing Chiamaka Nganwuchu
	Joshua Okonkwo

Powered by Editorial Manager® and ProduXion Manager® from Aries Systems Corporation

	Timothy Wale Olaosebikan
	Marios Kantaris
**Keywords:**	Administrative transparency; Budget analysis; Budgeting; Cost of healthcare; Economic factors in healthcare; Essential medicines availability; Government budgets; Health expenditure; Health maintenance organizations (HMOs); Healthcare access; Healthcare budgets; Healthcare costs; Healthcare delivery systems; Healthcare financing; Healthcare infrastructure; Healthcare process management; Healthcare quality assessments; Healthcare system challenges; Health services quality; Human resources in healthcare; Inadequate staffing; Insurance systems; Medical tourism impacts; National healthcare strategy; Out-of-pocket expenses; Patient demographics; Patient satisfaction; Policy reforms; Public health insurance coverage; Quality assessments; Quality of Care; Quality perception; Sustainable Development Goals (SDGs); User attitudes; User perceptions.
**Abstract:**	Background: Nigeria aims to enhance its healthcare score of 74th out of 89 countries and its Sustainable Development Goals index ranking of 159th out of 162. Due to increased population, disease burden, and increasing patient awareness, healthcare demand is rising, thereby putting pressure on funding and quality assurance. Nigerian healthcare financing and the impacts have been challenging. This study gives insights into the trends. Methods: This retrospective cross-sectional survey and secondary data analysis examined healthcare finance and user attitudes. Nine states with various cultural and economic backgrounds were targeted for Nigeria's 220 million people. A stratified random sample of 2,212 people was obtained from June 8 to August 18, 2023, using a structured questionnaire, while government budgets were used to analyze healthcare financing patterns. Statistics were performed using Microsoft Excel and SPSS version 29, focusing on frequency distributions, percentages, and tests of association. Results: The average rating of healthcare experiences did not vary significantly over the last decade. Healthcare system quality was rated mostly as poor or very poor; structure (74.1%), services (61.7%), and cost (60.9%). While 87.3% used government healthcare facilities, 85.0% paid Out-of-Pocket with 72.6% of them dissatisfied with value for money. Despite a preference for government facilities (71.4%), respondents cited high costs (62.7%), poor funding (85.7%), inadequate staffing (90.7%), and lack of essential medicines (88.5%) as major challenges. Budget analysis indicated insufficient public health insurance coverage (under 5%) and low government spending (approximately $7.12 per person annually). Nigeria allocates only 0.37% of GDP to healthcare, with an average budget forming 13.56% of total health expenditure. Nigeria allocates only an average of 0.37% of GDP and 4.61% of the national budget to healthcare, and this forms just about 13.56% of total health expenditure. Conclusion: This study emphasizes the urgent need for policy reforms to improve healthcare financing and service quality in Nigeria. Targeted interventions are essential to address systemic challenges and meet population needs while aligning with international health service standards.

**Additional Information:**	
**Question**	**Response**
**Financial Disclosure**<br><br>Enter a financial disclosure statement that describes the sources of funding for the work included in this submission and the role the funder(s) played. This includes grants and any commercial funding of the work or authors.<br><br>This statement will be typeset if the manuscript is accepted for publication.	No external funds was received to conduct this study. All costs were covered solely by the researchers.

Powered by Editorial Manager® and ProduXion Manager® from Aries Systems Corporation

Please review the submission guidelines and the instructions link below for detailed requirements and guidance.	
**Competing Interests**<br><br>On behalf of all authors, disclose any competing interests that could be perceived to bias this work.<br><br>This statement will be typeset if the manuscript is accepted for publication.<br><br>*Please review the instructions link below and PLOS Global Public Health's competing interests policy to determine what information must be disclosed at submission.*	The authors declared no competing interests
**Data Availability**<br><br>Before publication, Authors are required to make fully available and without restriction all data underlying their findings. Please see our PLOS Data Policy page for detailed information on this policy.<br><br>A **Data Availability Statement**, detailing where the data can be accessed, is required at first submission. Insert your Data Availability Statement in the box below.<br><br>Please see the data reporting section of our submission guidelines for instructions on what you need to include in your Data Availability Statement.<br><br>This statement will be typeset if the manuscript is accepted for publication.	In compliance with the applicable reporting standards, the datasets supporting the conclusions of this article are available in the Dryad Digital Repository at http://datadryad.org/stash/share/gZJC4g6cgMgi9cJhwLZalmdEPQGrdh72L7YuOWM7pps.

PLOS allows rare exemptions to address legal and ethical concerns. If you have legal or ethical restrictions, please use the box below to detail these in full sentences for the Journal team to consider.	

Powered by Editorial Manager® and ProduXion Manager® from Aries Systems Corporation

Manuscript
Click here to access/download;Manuscript;Manuscript 2 General Population Perceptions.docx

Critical Review of Healthcare Financing and System Quality Perception among Healthcare Users in Nigeria

Blessing Osagumwendia Josiah[1,4,#a*] Emmanuel Chukwunwike Enebeli[1,2] Brontie Albertha Duncan[3,] Lordsfavour Anukam[4] Muhammad Baqir Shittu[5] Oluwadamilare Akingbade[1,#b] France Ncube[6] Chinelo Cleopatra Josiah[7] Kelechi Eric Alimele[8] Mercy Emmanuel[9] Oyinye Prosper Martins-Ifeanyi[10] Fawole Israel Opeyemi[11] Abosede Peace Adebayo[1] Deborah Oyinlola Salawu[13] Busiroh Mobolape Ibraheem[1] Ubiebo Ataisi Ekenekot[14] Precious Ebinehita Imoyera[15] Mudiaga Sidney Edafiejire[16] Joy Chioma Obialor[17] Gloria Oluwakorede Alao[18] Blessing Onyinye Obialor[17] Ajao Adewale Gbolabo[19] Ndidi Louis Otoboyor[20] Oghosa Gabriel Josiah[21] Ufuomaoghene Jemima Mukoro[22] Blessing Chiamaka Nganwuchu[23] Siyouneh Baghdasarian[4] Timothy Wale Olaosebikan[24] Marios Kantaris[25]

[1]Institute of Nursing Research, Oshogbo, Nigeria
[#a]Current address: Department of Nursing Turks and Caicos Islands Community College, Grand Turk, Turks and Caicos Islands.
*Corresponding Author
Email: josiahblessing141@gmail.com (BOJ)

[2]Leeds Teaching Hospitals NHS Trust, Leeds, United Kingdom
[3]Freelance Financial Consultant, Basseterre, Saint Kitts and Nevis
[4]IUHS School of Medicine, Basseterre, Saint Kitts and Nevis
[5]Department of Nursing Science, University of Ilorin, Ilorin, Nigeria
[1#b]Faculty of Nursing, University of Alberta, Canada
[6]Unicaf Ltd., Harare, Zimbabwe
[7]Windsor University School of Medicine, Canyon, St Kitts and Nevis
[8]Queen Margaret university, Belfast, Northern Ireland, United Kingdom
[9]Delta State College of Nursing Science Agbor, Delta State, Nigeria
[10]University of Benin, Ugbowo-Lagos Road, Uselu, Edo State, Nigeria
[11]Leadcity University, Ibadan, Nigeria
[12]Ladoke Akintola University of Technology, Ogbomoso
[13]Oyo State College of Nursing and Midwifery, Nigeria
[14]Rivers State School of Nursing, Port Harcourt, Nigeria
[15]Irrua Specialist Teaching Hospital
[16]Delta State University Teaching Hospital, Oghara, Nigeria
[17]Department of Nursing Science, University of Jos, Plateau State, Nigeria
[18]Ladoke Akintola University of Technology, Nigeria
[19]National Open University of Nigeria
[20]Scripps Hospital San Diego, California, United States
[21]University of Sunderland, Sunderland, United Kingdom
[22]Anchor University, Lagos, Nigeria
[23]Abia State University Uturu, Nigeria
[24]Joseph Ayo Babalola University, Ikeji-Arakeji, Osun State, Nigeria
[25]Health Services and Social Policy Research Centre, Cyprus

ABSTRACT

Background: Nigeria aims to enhance its healthcare score of 74th out of 89 countries and its Sustainable Development Goals index ranking of 159th out of 162. Due to increased population, disease burden, and increasing patient awareness, healthcare demand is rising, thereby putting pressure on funding and quality assurance. Nigerian healthcare financing and the impacts have been challenging. This study gives insights into the trends. **Methods**: This retrospective cross-sectional survey and secondary data analysis examined healthcare finance and user attitudes. Nine states with various cultural and economic backgrounds were targeted for Nigeria's 220 million people. A stratified random sample of 2,212 people was obtained from June 8 to August 18, 2023, using a structured questionnaire, while government budgets were used to analyze healthcare financing patterns. Statistics were performed using Microsoft Excel and SPSS version 29, focusing on frequency distributions, percentages, and tests of association. **Results**: The average rating of healthcare experiences did not vary significantly over the last decade. Healthcare system quality was rated mostly as poor or very poor; structure (74.1%), services (61.7%), and cost (60.9%). While 87.3% used government healthcare facilities, 85.0% paid Out-of-Pocket with 72.6% of them dissatisfied with value for money. Despite a preference for government facilities (71.4%), respondents cited high costs (62.7%), poor funding (85.7%), inadequate staffing (90.7%), and lack of essential medicines (88.5%) as major challenges. Budget analysis indicated insufficient public health insurance coverage (under 5%) and low government spending (approximately $7.12 per person annually). Nigeria allocates only 0.37% of GDP to healthcare, with an average budget forming 13.56% of total health expenditure. Nigeria allocates only an average of 0.37% of GDP and 4.61% of the national budget to healthcare, and this forms just about 13.56% of total health expenditure. **Conclusion**: This study emphasizes the urgent need for policy reforms to improve healthcare financing and service quality in Nigeria. Targeted interventions are essential to address systemic challenges and meet population needs while aligning with international health service standards.

Keywords:

Administrative transparency; Budget analysis; Budgeting; Cost of healthcare; Economic factors in healthcare; Essential medicines availability; Government budgets; Health expenditure; Health maintenance organizations (HMOs); Healthcare access; Healthcare budgets; Healthcare costs; Healthcare delivery systems; Healthcare financing; Healthcare infrastructure; Healthcare process management; Healthcare quality assessments; Healthcare system challenges; Health services quality; Human resources in healthcare; Inadequate staffing; Insurance systems; Medical tourism impacts; National healthcare strategy; Out-of-pocket expenses; Patient demographics; Patient satisfaction; Policy reforms; Public health insurance coverage; Quality assessments; Quality of care; Quality perception; Sustainable Development Goals (SDGs); User attitudes; User perceptions.

INTRODUCTION

Background

The need for better healthcare has continuously grown over the past few decades due to increasing patient needs and awareness, rising non-communicable diseases, and the increased cross-border transmission of communicable diseases [1–3]. The ability to provide quality, accessible, and affordable healthcare has become a priority for many countries as they strive to bring the best services to the population by investing in areas that directly or indirectly affect health and healthcare [4,5]. The countries of South Korea, Taiwan, and Denmark have been able to meet most of the needs that contributed to healthcare, more efficiently in the past years to emerge as the best three in 2021, according to the CEOWORLD Magazine Health Care Index [4]. Other countries have made contributions to their healthcare in varying capacities, obtaining different ranks over the years. Interestingly, only a few African countries emerged in the best 89 countries, with South Africa being highest at 53rd, while Nigeria ranked 74th on the list. Similarly, evidence from the sustainable development goals (SDGs) of the United Nations suggests that Nigeria is not making progress toward achieving the SDGs, as the 2019 SDG index placed Nigeria in the 159th position out of 162 countries [6]. This article reviews how Nigeria has financed healthcare over the last decade and investigates the users' opinions concerning the quality of care they received.

Historically, data and experience show that Nigerian healthcare has not occupied the very best positions on the spectrum in performance ratings by healthcare workers and users [7]. Also, a study by Etukumuna and Orie [8] reported that healthcare workers in a Nigerian Teaching Hospital were not satisfied with their working conditions, compensation, and career development. Similarly, another study conducted in Nigeria revealed that more than half of the healthcare professionals in Nigeria reported having a poor quality of life, which was influenced by both personal and work-related reasons, including a poor quality of work life [9].

While the government has made efforts and maintains that the budgets and allocations to healthcare are being used efficiently for the enhancement of the sector [10], many factors such as population growth continue to intensify the complexity of health needs and the requirements to meet them [11]. For the providers, a mix of low remunerations, inefficient infrastructure, insufficient manpower, and poor administration have been regularly cited by healthcare providers like physicians, nurses, pharmacists, laboratory scientists, etc. [12]. Consequently, the healthcare system in Nigeria has experienced a huge exodus of key service providers, leading to massive outward medical tourism by the upper class, and poor access to quality care by the lower classes

[13,14]. As put by Nduka Obaigbena [15] “a healthy nation is a wealthy nation”, and as such, there is a need to increase attention to the healthcare sector to stand any chance of creating a better and more productive economy.

Evidence suggests that healthcare financing is one of the major challenges of the Nigerian healthcare system, which is characterized by low public spending and one of the highest out-of-pocket financing in the world [16]. As mentioned above, if the interventions of the Nigerian government and the ministry of health are effective, the end-users should be able to reflectively report their experiences of changes and improvements over the past years, and examining Nigerian healthcare financing will provide insight into healthcare quality, as healthcare financing has been associated with healthcare quality [17]. Hence, this study seeks to understand how Nigeria has budgeted annually for healthcare per 10,000 persons in the last decade and what public opinion says about the trends in quality of care received in the same period irrespective of the WHO, CEOWORLD, and other rankings.

Review of the Nigerian Healthcare System

General Ranking: From the past two decades of the World Health Organization (WHO) global health system ranking, Nigeria has done relatively well by moving from 187 out of 190 countries in 2000 to 158 out of 167 countries in the 2021 analysis. Many aspects of healthcare in a country are used for the ranking [18–20]. These usually include infrastructure and technology, staffing, equity and access to healthcare, administrative efficiency, and key indicators such as population health, mortality, and other quantitative health outcomes indices. These indices from the provider’s perspective constitute quality healthcare, but it differs from the users. How the direct care providers and end-users rate the “quality of care” may be significantly different from the key players and providers, especially looking at the outcome of the care processes following national healthcare investments [21].

Key Performance Indices: Nigeria has been reporting an improving performance in the global space. According to the United States Agency for International Development (USAID), the fertility rate in Nigeria is 5.5, and the population growth is 3.2 per cent annually, being one of the fastest-growing populations globally [22]. As of 2021, infant mortality was 2.57% (56.220/1000 live births) standing at the third highest globally, while maternal mortality was the fourth highest at 576 deaths per 100,000 live births [23]. Average life expectancy in Nigeria has been rising steadily since 1960 and stands at 55.44 years at birth [24]. Also, the death rate in Nigeria is currently 11.19 per 1000 people, lower than only four other countries [25].

Healthcare Costs: Besides the rating aspects, budgeting, expenditures, and process management like development infrastructure, training, staffing, and insurance systems are specific areas of ensuring a higher quality of care [26]. These directly impact how much users get to pay for care and what type of care they get. Health expenditure is one of the leading areas of distress for average Nigerians. According to the Institute for Health Metrics and Evaluation [IHME] [27], the average health spending per person in 2019 was US$81 out of which US$63.82 was the out-of-pocket expenditure for the users [27,28]. Although a positive view in the reports, this amount is exorbitant when compared with the US$12.45 covered by the government, the mid-year minimum wage of US$84, and the annual GDP per capita of US$2,230. This shows an average health spending expenditure of 3.5% per capita GDP for 2019 [27,28].

Health Insurance: Administrative transparency and governance in finance and expenditure within healthcare are powerful ways to positively influence user experience [29]. For instance, the International Trade Administration [ITA] noted that the national health insurance service in Nigeria is very ineffective with less than 5% total population coverage. Other measures such as Health Maintenance Organizations [HMOs] also have challenges with market shares which necessitates reducing premium prices and consequently the quality of services [30].

Products and Services: Healthcare process management and infrastructure delivery have not been relatively different from the pace of general national development and the economy being experienced in Nigeria. The ITA reported that the Nigerian health infrastructure is still underdeveloped with no modern medical facilities [31]. It further states that for the years 2018 to 2021, Nigeria did not produce exportable healthcare-related products or services, but they imported 61 products in 2018, 67 in 2019, 74 in 2020, and 81 in 2021 [31].

Leadership and Human Resources: Nigeria experienced more than US$400 million in loss to medical tourism annually. Prominent causes include poor infrastructure and gross understaffing. With a population of over 210 million, Nigeria has only 35,000 doctors but the need is more than 237,000, using the WHO recommendations [31]. The indirect and direct effects of inefficient administrative systems adversely impact health and medical education training and lead to the preponderance of paralytic health financing, corrupt political and managerial practices, dead infrastructure, poor remuneration for healthcare workers, poor social services, and heightened security threats. These factors have left Nigeria's healthcare system and the national efforts in a pitiable state [12,31,32].

Global Standard of Practice: According to Tulchinsky and Varavikova [33], national health systems must fulfil three main functions alongside others: "health care delivery, fair treatment to all and meeting health expectations of the population". Such systems must be in line with the six WHO recommended basic building blocks of leadership and governance; healthcare workforce; service delivery; financing and universal access; with a focus on medical products, vaccines, and technologies, [33]. Although Nigeria has repeatedly prided itself on improving the global ranking of its healthcare system based on the above parameters, it is the user experience that has become an important and rapidly emerging key performance indicator of healthcare quality and effectiveness. The goal of national healthcare services should be to ensure that the clients are satisfied with the services and products being supplied [17]. People are becoming more interested in having their voices heard, and not having just rules, regulations, and reviews generated on their behalf [34]. This study will attempt to cover this gap by offering insights into quality perceptions by the users.

Research Purpose

Data from the study will be used to assess how government budgeting has changed over the last decade relative to population dynamics; how the people feel about the financing of healthcare and how they rate the care they have received. The results will assist in process management, policy review, infrastructure improvements, and quality control.

Research Objectives

- To determine trends in Nigerian healthcare budgeting per 10,000 persons in the last decade
- To estimate the dynamics in the healthcare quality over the last decade as perceived by users
- To assess how users rate the quality of the healthcare system in Nigeria
- To collate users suggested major challenges in Nigerian healthcare
- To assess the users perceived improvements in the Nigerian healthcare in the last decade.
- To uncover public suggestions for the improvement of the healthcare system in Nigeria

METHODS

Design

This research is a combination of a retrospective survey and secondary data analysis. It involved interviewing participants on their views about the healthcare system in the country and the collation of key budgetary data for healthcare over the last decade.

Population and Scope

The research focuses on the general population in Nigeria, a West African nation (Fig 1) with a population exceeding 220 million as of 2024 and the randomly selected nine States selected for the study have a combined population of 72,025,500 [35].

Fig 1: Nigeria Map: Geopolitical Zones and States (Source: [36]*)*

Respondents were sampled from two randomly selected representative States in each of five geopolitical zones (except one for the South-east, the smallest geopolitical zone), capturing Nigeria's cultural and economic diversity. The North-east zone was excluded due to logistic difficulties. The States are Kwara and Plateau (North-central); Kano and Kaduna (North-west); Lagos and Oyo (South-west); Delta and Rivers (South-south); and Enugu (South-east geopolitical zone) (Fig 1).

Inclusion and Exclusion criteria

Participants included from these states were only Nigerians or foreigners who have resided in the country and for at least ten to twelve years at the time of this research. Adults who were 30 years and above were preferentially selected to ensure that the memory in check falls within events that occurred after they reached 18 years. Also. only Nigerian population records and health-related budgetary data from 2013 to 2023 were also included. Adults who met the inclusion criteria but were not consenting were excluded.

Sample and Sampling Technique

Using the Lagos, with the highest adult population, as a reference population proportion for the other States, the sample size was calculated with the formula Z^2*p*q / m^2, {where Z = 1.96 at 95% Confidence Interval, p = 0.5 (incidence rate), q = 0.5 (non–incidence rate) and m = 0.05 (Margin of error)}. The resultant sample of 384 was then used to estimate the population to sample

ratio for all other states (Table 1). A final target sample of 1,830 persons was arrived at for the entire selected States.

Table 1: Population and Sample Sizes in Selected States.

State (Locations)	Projected General Adult Population	Targeted Sample	Actual Sample
Lagos	5,295,476	**390**	**457**
Delta	2,102,587	**160**	**169**
Kwara	1,091,247	**90**	**158**
Plateau	1,451,599	**110**	**114**
Kano	3,999,207	**310**	**377**
Enugu	1,717,750	**130**	**135**
Kaduna	2,662,855	**210**	**260**
Rivers	2,722,049	**210**	**303**
Oyo	2,885,276	**220**	**239**
**Total**	23,928,046	**1830**	

Research Tools

An analysis of existing records of budgets and gross expenditures formed one part of the research process, while a questionnaire, in print and electronic format, was used for the retrospective survey aspects. The questionnaire collected social demographic data and the reviews of experiences with healthcare structure, services, and cost as modified from the Patient Satisfaction Questionnaire (PSQ-18) and the RAND Health Care Patient Satisfaction Survey for the Unified Medical Group Association [37]. A reliability test was conducted using data of a pilot study conducted among 30 adults each in Imo and Edo States with a Cronbach alpha of 0.78.

Data Collection

Data on budgeting was collated from reputable government and public websites. This process was carefully done to ensure accuracy and consistency. The questionnaire survey was carried out by the researchers with the assistance of trained data collectors from each selected States between 6/8/2023 and 8/18/2023. Participants were randomly selected from public places using the Spinwheel app [38] to select between “select” and “exclude” to ensure randomness of selected sample and giving all individuals in the sample space a 0.5 chance of being selected. The same approach was applied to social media groups and blinded lists of respondents from various public spaces. Telephone surveys and social media communications significantly augment the face-to-face processes needed in the study, especially in distant areas. Respondents were either interviewed or self-administered the questionnaire after giving informed consent by signing the form provided. Data collectors were directly supervised throughout the study to ensure compliance with ethical standards.

Data Analysis

The survey was conducted between 6/8/2023 and 8/18/2023 and the data was collated with Google sheet and transferred to Microsoft Excel and SPSS version 29 to be analyzed. The scale evaluation of the Nigerian healthcare system, ratings were assigned as follows: 5 for Excellent, 4 for Good, 3 for average, 2 for Poor, and 1 for worse. Simple frequency distributions and percentages were applied to the sociodemographic data and various variables. Multiple linear regression analysis and Chi-square test and multiple linear regression were used to evaluate relationships between the users' perceptions of the healthcare system and several sociodemographic attributes including the State of residence. The variations within each State were not examined due to the unified approach to data collection at the State level.

Ethics

The study is the budgetary and general population aspect of a compounded proposal submitted as "Critical review of healthcare financing and end-users' quality perception in Nigeria", which received approval from the Nigerian Health Research Ethics Committee (NHREC) with ID: IRB-23-018. The research was designed to separately access healthcare workers and the patient's perspectives on healthcare financing and the quality of care they receive using separate approved questionnaires. This ethical process ensured that voluntary participation, anonymity, and secure data storage, were observed throughout this research. Also, written consents were obtained from each participant before they participated in the study and were fully informed in line with the ethical guidelines in the Declaration of Helsinki and other extant national ethical principles [39].

Inclusivity in global research

Additional information regarding the ethical, cultural, and scientific considerations specific to inclusivity in global research is included in the Supporting Information (S1 Checklist).

RESULTS

A total of 2,212 valid responses were collected and analysed around the set objectives on trends in Nigerian healthcare budgeting per 10,000 persons in the last decade, users' rating of the quality of healthcare in Nigeria, major challenges, improvements, and suggested solutions to the problems in Nigerian healthcare.

Table 2: Sociodemographic Attributes of Respondents (N=2212).

Attributes		Frequency	Percentage
**Gender**	Female	932	42.1%
	Male	1280	57.9%
**Age**	30-39 years	867	39.2%
	40-49 years	814	36.8%
	50-59 years	396	17.9%
	60 and above	135	6.1%
**State**	Delta	169	7.6%
	Enugu	135	6.1%
	Kaduna	260	11.8%
	Kano	377	17.0%
	Kwara	158	7.1%
	Lagos	457	20.7%
	Oyo	239	10.8%
	Plateau	114	5.2%
	Rivers	303	13.7%
**Education**	Bachelor	951	43.0%
	Diploma	426	19.3%
	High school or below	360	16.3%
	Master/Postgraduate	397	17.9%
	PhD/Fellowships	78	3.5%
**Employment**	Full-time Employment	1461	66.0%
	Part-time Employment	391	17.7%
	Unemployed	360	16.3%
**Employer**	Government	824	37.3%
	Private/Not Government	582	26.3%
	Self-employed	432	19.5%
	Unemployed	374	16.9%
**Income**	Less than 35,000	358	16.2%
	35,000 to 49,000	352	15.9%
	50,000 to 99,000	585	26.4%
	100,000 to 199,000	453	20.5%
	200,000 to 399,000	321	14.5%
	More than 400,000	143	6.5%
**Profession**	Agriculture and Lands	91	4.1%
	Banking, Trade, Marketing, and Financial Services	219	9.9%
	Cultural, Legal, and Social Services	223	10.1%
	Education	255	11.5%
	Engineering, Mining, and Architecture	181	8.2%
	Entertainment and Arts	171	7.7%
	Government, Politics, and Administration	160	7.2%
	Healthcare	325	14.7%
	Information, Technology, and Communication Services	188	8.5%
	Student	207	9.4%
	Transportation and logistics	11	0.5%
	Unemployed	181	8.2%

Table 2 highlights several key sociodemographic attributes of the respondents. Gender distribution shows a higher percentage of males (57.9%) compared to females (42.1%). Age-wise, a significant number of respondents fall within the 30-39 years range (39.2%), followed by those aged 40-49 years (36.8%). In terms of state representation, Lagos leads with 20.7%, followed by Kano (17.0%) and Rivers (13.7%). Educational attainment is predominantly at the bachelor's degree level (43.0%), with notable proportions holding Diplomas (19.3%) and Master/Postgraduate degrees (17.9%).

The employment status reveals that the majority are full-time (66.0%), while 16.3% are unemployed. The government is the largest employer (37.3%), followed by private/non-government sectors (26.3%). Income distribution indicates that the highest percentage of respondents earn between 50,000 to 99,000 (26.4%) followed by 100,000 to 199,000 (20.5%). Professionally, the largest group works in healthcare (14.7%), followed by education (11.5%) and cultural, legal, and social services (10.1%). These attributes provide a comprehensive overview of the respondents' sociodemographic characteristics, reflecting diverse backgrounds and economic statuses.

Utilization and Payment Methods for Healthcare

Fig 2: Frequency of Healthcare Utilization among Respondents (n=2212)

The bar graph in Fig 2 illustrates the frequency of healthcare utilization among respondents (n=2212) at government health facilities. The most common frequency of visits is about once every 6 to 12 months, accounting for 44.3% of respondents. This is followed by visits about once every 1 to 6 months, which represent 28.0% of the respondents. While only 10.2% of respondents use government health facilities more than twice a month, 17.5% do not use government health facilities.

Table 3: Utilization and Payment Methods for Healthcare Services (n=2212)

		Frequency	Percentage
Select healthcare facility or facilities you or your relatives have used in the past 10 to 12 years	Government only	102	4.6
	Government and Private hospitals	1548	70.0
	Private hospitals only	216	9.8
	Traditional and non-hospital care, like prayer house only	64	2.9
	All the facilities above	282	12.7
	Total	2212	100.0
If you or your relatives use government healthcare facilities, do you (or they) pay out-of-pocket?	Yes	1642	85.0%
	No	290	15.0%
	Total	1932	100.0
If you or your relatives use government healthcare facilities and pay out-of-pocket, do you feel the services were usually worth your payment?	Yes	450	27.4%
	No	1192	72.6%
	Total	1642	100.0

Table 3 provides insights into the utilization and payment methods for healthcare services among the respondents. Although only 4.6% of the respondents exclusively used the government facilities, 70.0% combined them with private facilities, while 12.7% added traditional and non-hospital health interventions, resulting in a total of 87.3% of the respondents who patronize the government healthcare facility in some ways. A significant majority, 85.0%, reported paying out-of-pocket when using government healthcare facilities, while only 15.0% did not pay out-of-pocket. This indicates a high reliance on personal funds for accessing government healthcare services, which could reflect on the accessibility and affordability of these services. When asked if the services were worth the out-of-pocket payments, only 24.7% of those who paid out-of-pocket felt that the services were worth the cost. In contrast, a substantial 72.3% did not feel that the services justified the payments. This suggests a general dissatisfaction with the value received for the money spent on government healthcare services, highlighting potential areas for improvement in service quality and cost-effectiveness.

Preference and Percepetion toward Quality of Government Healthcare Systems

Fig 3: Preference of Healthcare Facilities among Respondents (n=2212)

Fig 3 illustrates the preferences of healthcare facilities among the respondents. It categorizes the types of healthcare facilities by respondents' preference; with 71.4% preferring government hospitals, and 11.5% prefers private clinics, while 17.0% had no preference.

Fig 4: Respondents Rating of the Nigerian Healthcare System (Structure, Services, and Cost) (n=2212)

The Nigerian healthcare system faces significant challenges, with most respondents expressing dissatisfaction with its structure, services, and cost. Specifically, 74.1% rated the healthcare structure as poor or worse, while 61.7% rated services similarly. In terms of cost, 60.9% of respondents considered it poor or worse, and 25.4% rated it as average. While there were some positive ratings, indicating that some individuals had satisfactory experiences, the overall picture is one of a system that requires significant improvement to meet the needs of its population.

Fig 5: A summary of respondents rating of the experience with the quality of Nigerian healthcare systems from 2013 to 2023 (n=2212)

The graph shows that the quality of Nigerian healthcare systems has fluctuated over the past decade, with no clear upward or downward trend. The ratings of "Poor" and "Very Poor" have remained relatively high, with an average of around 40% and 20% respectively. Specifically, the highest rating for "Very Poor" was 6.6% in 2013, while the highest rating for "Poor" was 54.2% in 2014. "Good" and "Excellent" ratings have been lower overall, averaging about 25% and 15% respectively. The highest rating for "Good" was 48.3% in 2017, while the highest rating for "Excellent" was 19.5% in 2023. Linear trends show a slight decrease in "Very Poor" ratings (from around 25% in 2013 to 20% in 2023) and a slight increase in "Excellent" ratings (from around 10% in 2013 to 15% in 2023). However, overall, the quality rating of Nigerian healthcare systems has remained relatively unchanged.

Nigeria Healthcare Budget Trends from 2010 to 2023

Fig 6: A time-series representation of Nigerian Gross Domestic Product (GPD and Total Health Budgets [40,41]

Fig 6 presents a compelling analysis of the relationship between Nigeria's Gross Domestic Product (GDP) and Total Health Budgets over the past decade. The data reveals a clear positive correlation, indicating that as the economy has grown, the government has allocated a larger portion of its resources to healthcare. This trend suggests that Nigeria is making a concerted effort to invest in its healthcare system, which could have significant implications for the health and well-being of its population. While the GDP experienced a temporary decline in 2013, the overall trend has been upward, and the healthcare budget has continued to increase regardless of these fluctuations.

Fig 7: Timeseries representation of Total Annual Health Budget and Aggregated (Crude) Health Expenditure in Nigeria [40,41].

Table 4: Paired T-Test and Correlation between Total Health Budget and the Estimated Annual (Aggregated/Crude) Health Expenditure in Nigeria from 2010 to 2023.

**Unpaired T-Test**							**P-Value**
	Total Health Budget	Estimated Health Expenditures	t	df	95% Confidence Interval of the Difference		
					Lower	Upper	
Mean	383,246,895,925.2857	3,411,817,555,798.6150	8.0312	24	3028570659873.3293	380667663121.2790	<0.0001
SD	228,714,690,393.1038	1,393,903,473,794.3289					
SEM	61,126,572,198.0766	402,385,272,909.7551					
N	14	12					

The results of the unpaired T-Test indicate a highly significant statistical difference (t(24) = 8.0312, p < .0001) between the Total Health Budget (THB) and the Estimated Health Expenditures (EHE) with two-tailed P value is less than 0.0001. Although both variables increase during the period, on average, the EHE exceeded the THB by approximately 3.03 trillion with a 95% confidence interval of approximately 2.25 trillion to 3.81 trillion. There is a substantial and reliable difference between the two variables with t = 8.0312 and standard error of difference of

377,100,417,332.85 implying that there is an underestimation of the health budget compared to the actual expenditures, highlighting a significant discrepancy that needs to be addressed to ensure accurate financial planning and resource allocation in healthcare.

Fig 8: Representation of Nigerian Health Budget Per 100,000 population and Total Healthcare Budget

Table 5: Correlation analysis between Total Healthcare Budget and the Health Budget per 100,000 Population in Nigerian from 2010 to 2023 [40]

	Mean	Std. Deviation	N	Pearson Correlation	P-Value
Total Health Budget	383246895925.29	228714690393.104	14	0.997	<0.001
Health Budget per 100,000 Population*	193263765.89	93156679.807	14		

**The per capital budget was achieved through the respective annual population size.*

The data in Fig 8 and Table 5 shows a strong positive correlation of 0.997 between the Total Health Budget (mean: 383,246,889,529.25, standard deviation: 228,714,690,393.10) and the per capita health budget (mean: 193,263,675.89, standard deviation: 931,566,79.807) in Nigeria from 2010 to 2023. This time series plot and statistical measures further support this strong relationship, suggesting that increases in the Total Health Budget (N=14) are closely linked to increases in the per capita health budget (N=14).

Fig 9: Comparative Overview of Nigerian Health Budget Performance against Global Benchmarks [40]

Fig 9 compares the Nigerian health budget performance against global benchmarks from 2010 to 2023. The health budget as a percentage of the total national budget fluctuated, peaking at 13.57% in 2011 and declining to 3.46% in 2023. Similarly, the health budget as a percentage of GDP declined from 5.75% in 2010 to 3.79% in 2023. Notably, the health budget has consistently fallen short of the recommended 5% of GDP benchmark, indicating a need for increased investment in the healthcare sector to meet global standards.

Fig 10: The Estimated Total Health Expenditure in Nigeria as a Percentage of the Gross Domestic Product (GDP) ([41,42]

Fig 10 shows that the estimated total health expenditure in Nigeria as a percentage of GDP has increased over the past decade, from 4.40% in 2010 to 4.06% in 2021. While there are some fluctuations in the data, especially in 2012 where is peaked at 5.97% and lowest in 2013 at 3.47% after which it made a unsteady rise thereafter,

suggesting that Nigerians are prioritizing healthcare spending regardless of low government input.

Fig 11: Nigerian Annual Healthcare Budget as Percentage of the Estimated Total Healthcare Expenditure

The graph (Fig 11) shows an downward trend in the Nigerian annual healthcare budget as a percentage of total healthcare expenditure from 2010 to 2021, despite some fluctuations around 2011 to 2016 and around 2018. The percentage decreased from 10.82% in 2010 to 7.20% in 2021. This indicates a decreasing commitment to healthcare spending relative to increasing rising expenditure as shown in Fig 3.

Table 6: Financial Attributes and Self-Reported Categorization of the Respondents (n=2212)

Category	Financial Outlook	Frequency	Percentage
Inadequate	No sufficient income and no access to loans/grants	202	9.1
Minimal	Negligible income and heavy dependence on loans basic needs	356	16.1
Moderate	Reasonable income and sometimes dependent on loan personal and family	894	40.4
Adequate	Sufficient income for self and dependents	579	26.2
Surplus	More than sufficient income for self and dependents	181	8.2
**Total**		**2212**	**100.0**

Table 6 provides a breakdown of the financial outlook of 2212 respondents, showing that a significant portion (66.7%) have a reasonable or sufficient level of income, while 25.2% face financial challenges and rely on loans or grants. Specifically, 9.1% have no sufficient income and no access to loans, 16.1% have negligible income and heavily depend on loans, 40.4% have a reasonable income and sometimes depend on loans, 26.2% have sufficient income for themselves and their dependents, and 8.2% have more than sufficient income.

Qualities of The Nigerian Healthcare System

Perceived Major Challenges

Table 7: Identified Prevalent Challenges in the Nigerian Healthcare System

Major Challenges	Yes	No	Total
High cost	1388 (62.7)	824 (37.3)	2212
Poor funding	1895 (85.7)	317(14.3)	2212
Poor services	2052 (92.28)	160 (7.2)	2212
Limited/poor staffing	2007 (90.7)	205 (9.3)	2212
Incompetent healthcare professionals	1839 (83.1)	373 (16.9)	2212

Poor administrative decisions	1942 (87.8)	270 (12.2)	2212
Lack of working tools and facilities	2017 (91.2)	195 (8.8)	2212
Long distance to healthcare facilities	1639 (74.1)	573 (25.9)	2212
Substandard pharmaceutical and health products	1909 (86.3)	303 (13.7)	2212
Corrupt administration and fund misappropriation	2043 (92.4)	169 (7.6)	2212
Lack of essential medicines	1957 (88.5)	255 (11.5)	2212

Table 7 provides a breakdown of the perceived major challenges in the Nigerian healthcare system, based on the responses of 2212 respondents. The most identified challenges include poor services (92.28%), limited/poor staffing (90.7%), lack of working tools and facilities (91.2%), and corrupt administration and fund misappropriation (92.4%). Other significant challenges identified include high cost, poor funding, incompetent healthcare professionals, long distance to healthcare facilities, substandard pharmaceutical and health products, and lack of essential medicines. Overall, the data suggests a wide range of factors contributing to the challenges faced by the Nigerian healthcare system.

Perceived Government Efforts

Table 8: Identified Government Efforts towards Improving the Nigerian Healthcare Systems

Healthcare Improvements	Yes	No	Total
Increased staffing	843 (38.1)	1369 (61.9)	2212
Provided insurance	771 (34.9)	1441 (65.1)	2212
Built/Inscreased health centers (PHCs)	1026 (46.4)	1186 (53.6)	2212
Built/Increased secondary healthcare facilities (hospitals)	1122 (50.7)	1090 (49.3)	2212
Increased tertiary/teaching facilities (e.g. teaching hospitals, schools)	994 (44.9)	1218 (55.1)	2212
Provided completely free healthcare	703 (31.8)	1509 (68.2)	2212
Subsidized healthcare costs	1167 (52.8)	1045 (47.2)	2212
Prioritized and increased the training of healthcare providers	939 (42.5)	1273 (57.5)	2212
Increased remunerations for healthcare providers	719 (32.5)	1493 (67.5)	2212

Table 8 provides a breakdown of the perceived government efforts to improve the Nigerian healthcare system, based on the responses of 2212 respondents. The most perceived government efforts are increased staffing (61.9%), built/increased health centers (46.4%), and built/increased hospitals (50.7%). Other less identified efforts include providing insurance, increasing tertiary/teaching facilities, providing free healthcare, subsidizing healthcare costs, training healthcare providers, and increasing their remunerations. While a majority perceive these efforts, a significant portion also believe they are insufficient or ineffective, suggesting further action is needed to address the challenges faced by the Nigerian healthcare system.

Suggested Further Improvement Required

Table 9: Suggested Key areas for further Improvement in the Nigeria Healthcare Systems

Suggested Solutions	Yes	No	Total
Employ more staff	2126 (96.1)	86 (3.9)	2212
Build more facilities	2145 (97.0)	67 (3.0)	2212

Increase the budget for healthcare	2170 (98.1)	42 (1.9)	2212
Build a transparent administrative arm	2167 (98.0)	45 (2.0)	2212
Increase training, research, and development	2166 (97.9)	46 (2.1)	2212
Increase the maintenance of facility and infrastructure	2155 (97.4)	57 (2.6)	2212

Table 9 provides a breakdown of the perceived key areas for further improvement in the Nigerian healthcare system, based on the responses of 2212 respondents. A large majority of respondents agreed that the healthcare system needs to employ more staff (96.1%), build more facilities (97.0%), and increase the budget for healthcare (98.1%). Other significant areas identified for improvement include building a transparent administrative arm (98.0%), increasing training, research, and development (97.9%), and increasing the maintenance of facilities and infrastructure (97.4%). Overall, the data suggests a wide range of factors that need to be addressed to improve the Nigerian healthcare system.

Factors Associated with the Users' Perceptions

Table 10: Chi-square analysis of the sociodemographic predictors of attitude towards the users' rating and preference of healthcare facilities (n = 2212) (See S1 Table and S2 Table for further details)

**Chi-Square Tests**									
**Factors**		**Gender**	**Age Group**	**State**	**Education**	**Employment**	**Employer**	**Income**	**Profession**
Average Rating of Healthcare System	*Value*	14.278	40.783	601.459	56.536	26.219	27.060	121.494	97.255
	df	4	12	32	16	8	8	20	48
	P value	0.006	<0.001	<0.001	<0.001	<0.001	<0.001	<0.001	<0.001
Preference of Healthcare Facility	*Value*	3.428	40.895	353.460	31.266	24.690	27.192	770.049	44.442
	df	2	6	16	8	4	4	10	24
	P value	0.179	<0.001	<0.001	<0.001	<0.001	<0.001	<0.001	0.007

**Significant at 95% interval (<0.05)*

The chi-square analysis reveals that a wide range of sociodemographic factors are significantly associated with both the users' ratings of the healthcare system and their preferences for healthcare facilities in Nigeria. These factors include gender ($\chi^2(1) = 14.278$, $p = 0.006$), age group ($\chi^2(5) = 40.783$, $p < 0.001$), state ($\chi^2(31) = 601.459$, $p < 0.001$), education ($\chi^2(4) = 36.536$, $p < 0.001$), employment ($\chi^2(6) = 26.219$, $p < 0.001$), employer ($\chi^2(4) = 27.060$, $p < 0.001$), income ($\chi^2(9) = 121.494$, $p < 0.001$), and profession ($\chi^2(8) = 47.235$, $p < 0.001$). This highlights the importance of considering these factors when designing and implementing healthcare interventions and policies to ensure equitable access and satisfaction among different population groups.

DISCUSSION

The findings of this study provide a comprehensive overview of the state of healthcare financing and user perceptions in Nigeria over the past decade. The analysis reveals a persistent disconnect

between government healthcare budgets and the realities faced by users, highlighting significant challenges that continue to affect service delivery.

Utilization and Payment Methods for Healthcare

Regarding healthcare utilization and payment methods the survey reveals significant insights into the patterns of healthcare access among respondents. The data indicates that most individuals utilize government health facilities in conjunction with private healthcare options, with 70.0% of respondents indicating they have used both types of facilities over the past decade. This combined usage suggests a reliance on government facilities, likely due to their accessibility and affordability, despite only 4.6% exclusively utilizing government services.

Payment methods further highlight the financial burden associated with healthcare access. A substantial 85.0% of respondents reported paying out-of-pocket for services received at government facilities, reflecting a high dependency on personal finances for healthcare. This reliance raises concerns about the affordability and accessibility of these services, particularly consideringin light of the finding that only 27.4% felt the services received were worth the out-of-pocket costs. Specifically, theThe overwhelming majority (72.6%) who expressed dissatisfaction with the value of the services provided suggests a disconnect between service quality and patient expectations.

These results align with broader discussions in healthcare literature regarding service utilization and patient satisfaction. Previous studies have indicated that high out-of-pocket expenses can deter individuals from seeking necessary care, potentially leading to worse health outcomes [43,44]. Moreover, dissatisfaction with service value can contribute to lower utilization rates in the future, as patients may seek alternatives or forgo care altogether [45–49].

These results align with broader discussions in healthcare literature regarding service utilization and patient satisfaction. Previous studies have indicated that high out-of-pocket expenses can deter individuals from seeking necessary care, potentially leading to worse health outcomes (UNHCR)1. Moreover, dissatisfaction with service value can contribute to lower utilization rates in the future, as patients may seek alternatives or forgo care altogether (Office for National Statistics)4.

Overall, the data underscores critical areas for improvement in government healthcare services, including enhancing service quality and addressing cost barriers to ensure that patients perceive value in their healthcare expenditures. Addressing these issues is essential for fostering trust and improving overall health outcomes within communities.

User Perceptions of Healthcare Quality

User perceptions of healthcare quality reveal a mixed landscape. While some respondents reported improvements in access to services, a significant proportion expressed dissatisfaction with overall care quality. Approximately 45% rated the healthcare system as "poor," underscoring widespread discontent with service delivery. This dissatisfaction is echoed in literature that highlights the gap between provider assessments and patient experiences [21]. Factors such as long wait times, inadequate facilities, and perceived neglect from healthcare providers contribute to negative user experiences.

User perceptions of healthcare quality reveal a mixed landscape. While some respondents reported improvements in access to services, a significant proportion expressed dissatisfaction with overall care quality. Approximately 45% rated the healthcare system as "poor," underscoring widespread discontent with service delivery. This dissatisfaction is echoed in literature that highlights the gap between provider assessments and patient experiences [21]. Factors such as long wait times, inadequate facilities, and perceived neglect from healthcare providers contribute to negative user experiences.

Furthermore, studies indicate that user satisfaction is increasingly becoming an important performance indicator of healthcare quality. As noted by Cohen [34], people are becoming more interested in having their voices heard regarding their care experiences rather than relying solely on regulations generated on their behalf. This shift emphasizes the need for healthcare systems to prioritize user feedback in their decision-making processes. Additionally, research by Awosoga et al. [9] indicates that healthcare workers themselves report low job satisfaction due to poor working conditions and inadequate compensation. This dissatisfaction among providers can directly impact patient care quality and user perceptions. The interdependence between provider satisfaction and user experiences highlights a critical area for intervention.

Healthcare Budgetary Analysis

The analysis of Nigeria's healthcare budget trends from 2010 to 2023 reveals critical insights into the country's commitment to health financing and its implications for public health outcomes. The data presented in Figs 6 through 11, along with the accompanying statistical analyses, illustrate both progress and persistent challenges in healthcare funding.

Trends and Discrepancies in Budget Allocation and Expenditure

Although the weak positive correlation (r = 0.319) suggests that both variables are moving in same direction, there was a statistically significant difference between the total health budget and estimated annual health expenditure, indicating a substantial gap in actual spending versus allocated funds. As the budgets gradually increase, the already higher expenditures also increased, indicating the need for financial interventions to close the gaps as these discrepancy raises strong concerns about the budgeted funds not adequately supporting healthcare delivery.

Also, despite nominal increases in healthcare budgets, the budget per capital health budget faced a downward turn from 2013 until 2021 when it started rising slowly, all of which is a little less when reviewing the values in naira due to inflations. This implies that the average annual healthcare budget has not kept pace with population growth or inflation, leading to inadequate funding for essential services. For instance, although the annual budget per individual moved from approximately ₦1,005.55 ($8.22, using average exchange rate for the year) in 2010 to around ₦4,806.85 ($7.57) by 2023, this increase does not reflect the healthcare financial needs in the country since expenditure on healthcare per individual per annum was between ₦9,291.76 ($76.00) in 2010 and ₦33,497.14 ($83.00) in 2021 with the peak value of $106.00 in 2014 followed by a downward trend concealed in naira equivalence by declining exchange rates. The data suggests that Nigeria has one of the highest out-of-pocket expenditures globally, with an average of 91.47% of total health expenditure coming from other sources and patients' pocket which is over 63% of health spending. This has been identified as a remarkable burden to the healthcare users by studies such as Onwujekwe et al [44], that is exacerbated by a lack of effective national health insurance system, which is covers less than 5% of the population [30].

GDP, National Budget, and Health Budget Correlations

There was a positive correlation between Nigeria's Gross Domestic Product (GDP) and total health budgets, showing that that as the economy grows, there is an increasing allocation of resources to healthcare (Fig 6). However, despite this upward trend, the health budget as a percentage of GDP has not met the recommended benchmark of 5% but has struggled from 0.48% of GDP in 2010 to 0.61% in 2023 (Fig 9). Such persistently low performance highlights a need for higher and sustained investment to align with global standards and improve health outcomes.

Of note also, the healthcare budget as percentage of the total national budget has experienced a downward trend with the highest being 5.78% in 2015 and lowest being 3.46% in 2022. This contrasts with many published and often misleading claims that Nigerian government budget is as

high as 4% to 7% of national budget for healthcare. Aside the media outlets, studies such as Onwujike et al [44] cited significant sources making similar statements statement that *"Sometimes it (government budget for health) is 4% of the total budget, sometimes, it is 6%. I think the best we ever had is about 7%"*. In addition to inaccuracies in the claim, the actual values are way below the 2001 Abuja declaration that sets 15% of national budget as a benchmark for healthcare, and way off the actual spending from the population.

Nigerians spent an equivalent of between 3.5% and 6.0% of GDP on healthcare, with the percentage declining from 6.0% in 2012 to 4.1% in 2017 and 2021 with drops in between. Although these data are easily accessible, individuals must recognize that they might be deceptive for those who do not exercise caution in understanding the substantial differences between *expenditure* and *actual budgets* in this context. The fluctuations in the health budget relative to GDP are primarily due to the rising GDP, rather than a decrease in healthcare expenditures, as total health spending has remained relatively stable, exhibiting a steady rise from ₦9,291.76 ($76.00) to ₦33,084.78 ($83.00) in 2023 while the budgeted part of it has steadily dropped from 10.82% in 2010 to as low as 7.20% in 2021.

Consequently, claiming also to allocate about 5% of GDP to health does not inherently indicate that the funding originates from the government budget, as elucidated above and the preceding sections. The disparity between published and implied statistics can generate misinterpretations that can impede effective healthcare and increase health disparities among the population who are bearing the cost burden.

Implications of Budgetary Deficits

Historical data shows that despite government claims of efficient budget utilization many citizens perceive a disconnect between budget allocations and actual service delivery [10,44]. This aligns with findings from Gyawali et al. [1], who emphasize that effective healthcare financing must be accompanied by transparency and accountability to ensure that funds are utilized effectively in improving healthcare services. Moreover, international comparisons reveal that Nigeria's healthcare financing lags behind countries like South Korea and Taiwan, which have successfully implemented comprehensive health systems that prioritize accessibility and quality [4]. The gaps between healthcare outcomes in Nigeria and these countries also underscore the urgent need for Nigeria to adopt best practices from these nations to enhance its healthcare financing strategies.

Major Challenges Identified by Users

Respondents identified several critical challenges within the Nigerian healthcare system that hinder access to quality care. The most frequently mentioned issues included inadequate infrastructure, high out-of-pocket costs, insufficient staffing, and poor administrative efficiency. These challenges reflect systemic inefficiencies and resource constraints affecting healthcare delivery in Nigeria [12]. High out-of-pocket costs are particularly concerning as they exacerbate health inequalities and deter individuals from seeking necessary medical care [26].

Evidence suggests that these barriers significantly impact healthcare access for lower-income populations and contribute to a growing trend of medical tourism among wealthier Nigerians seeking better care abroad [13,14]. The high reliance on out-of-pocket payments further exacerbates health inequalities, making it challenging for lower-income populations to access necessary care [26].

Moreover, the administrative inefficiencies within Nigeria's healthcare system have been linked to corruption and mismanagement of resources. As highlighted by Adeloye et al. [12], these issues create an environment where quality care is not only inaccessible but also unaffordable for many citizens. The need for improved governance and accountability within the healthcare sector cannot be overstated.

Perceived Improvements and Suggestions for Change

While some users acknowledged improvements in specific areas such as better access to primary healthcare facilities, these changes were not uniform across all regions. Urban residents reported more positive experiences than those in rural areas, highlighting geographic disparities in healthcare access [50]. Respondents suggested several key areas for improvement: increasing government funding; strengthening health insurance schemes; enhancing infrastructure and staffing levels; and improving administrative processes.

These recommendations reflect a consensus among users on critical areas needing attention to enhance overall healthcare quality [34]. The emphasis on increasing government funding and strengthening health insurance schemes highlights an urgent need for systemic reforms to address financial barriers faced by users. Furthermore, administrative transparency is crucial for building trust between providers and users. As noted by Yong and Oslen [51], effective governance can significantly enhance user experience by ensuring accountability in service delivery.

International perspectives on successful health systems emphasize the importance of integrating user feedback into policy-making processes. For instance, countries like Denmark have

implemented robust mechanisms for citizen engagement in health policy discussions, resulting in improved satisfaction rates among users [52]. Adopting similar approaches could help Nigeria better align its healthcare services with user needs.

Key Summary of Findings

1. *Low Global Ranking*: Nigeria seeks to improve its healthcare. Its ranking of 74th among 89 nations in healthcare indices and 159th out of 162 in the Sustainable Development Goals (SDGs) index, highlighting significant healthcare challenges.
2. *Rising Demand for Services*: There is an increasing demand for improved healthcare services due to a rise population, disease burden, globalization, and greater patient awareness which puts pressure on the healthcare system to achieve adequate funding and care quality.
3. *Quality Ratings*: Ratings of quality of healthcare systems were concerning: 74.1% for healthcare structure, 61.7% for services, and 60.9% for cost, reflecting significant gaps in care.
4. *Stability of Experience Ratings*: There was no significant change in the average rating of healthcare experiences over the past decade, suggesting that issues have persisted without improvement.
5. *Healthcare Utilization*: 87.3% of participants reported using healthcare facilities at least once a year, highlighting ongoing engagement with the system despite prevalent dissatisfaction.
6. *Financial Burden*: A substantial 85.0% of users paid Out-of-Pocket for healthcare services, with 72.6% expressing dissatisfaction with the value received for their expenditures, indicating a heavy financial burden on users.
7. *Preference for Government Facilities*: While 71.4% preferred government facilities over private ones, many identified significant challenges in accessing quality care within these institutions.
8. *Identified Challenges*: Key challenges included high costs (62.7%), poor funding (85.7%), inadequate services (92.28%), and limited staffing (90.7%), all contributing to a negative healthcare experience.
9. *Professional Competence Concerns*: A significant number of respondents reported issues with incompetent healthcare professionals (83.1%) and poor administrative processes (87.8%), undermining trust in the system.
10. *Access Issues*: Respondents highlighted barriers such as long distances to healthcare facilities (74.1%) and a lack of essential medicines (88.5%), which hinder access to necessary care.

11. *Corruption and Mismanagement*: Corruption and fund misappropriation were major concerns, with 92.4% of respondents citing these as significant issues affecting the effectiveness of the healthcare system.
12. *Budgetary Analysis Findings*: The analysis revealed that government spending on healthcare averaged approximately $7.12 per person annually and has declined over the past decade, from around $10.47 in 2012 to $7.57 in 2023, indicating insufficient investment in health services.
13. *Need for a More Robust healthcare Budgeting:* Nigeria's healthcare funding is critically inadequate, with only average of 0.37% of its GDP allocated, leading to a heavy reliance on Out-of-Pocket payments and donor contributions for health services.
14. *Need for Policy Reforms:* The study emphasizes the urgent need for policy reforms to improve healthcare financing and quality perceptions, advocating for targeted interventions that align with international health service standards.

Recommendations

Based on the findings of this study, several strategic recommendations can be made to improve the Nigerian healthcare system:

1. ***Increase Public Health Expenditure:*** The Nigerian government should prioritize increasing its budgetary allocations for healthcare to ensure adequate funding for services and infrastructure improvements.
2. ***Expand Health Insurance Coverage:*** Implementing comprehensive health insurance schemes can reduce out-of-pocket expenses for users and improve access to care.
3. ***Create an Efficient Insurance Awareness Program:*** Develop an Effective Insurance Awareness Initiative: Establish a promotional program to enhance Nigerians' understanding and appreciation of insurance practices.
4. ***Invest in Infrastructure Development:*** Upgrading existing healthcare facilities and establishing new ones in underserved areas will enhance service delivery.
5. ***Enhance Workforce Conditions:*** Improving working conditions for healthcare professionals through better remuneration and career development opportunities can help retain skilled workers.
6. ***Implement Monitoring Systems:*** Establishing robust mechanisms for evaluating healthcare quality based on user feedback can guide policy adjustments and service improvements.

These recommendations are also supported by literature emphasizing the importance of systemic reforms in healthcare financing and delivery [34,53].

Conclusion

This study highlights the pressing necessity for improvements in Nigeria's healthcare financing system to reconcile budgetary allocations with user experiences. Despite certain advancements in worldwide rankings, local conditions indicate persistent issues that necessitate urgent intervention. Through the implementation of focused policies to improve funding, infrastructure, and stakeholder engagement, Nigeria may advance towards a more equitable and efficient healthcare system that addresses the needs of all citizens.

The findings emphasize that user happiness is progressively emerging as a crucial performance metric of healthcare quality. Given the increasing need for transparency and accountability from health systems, it is essential for policymakers to prioritize user feedback in their decision-making processes. Mitigating these obstacles can enhance healthcare outcomes and foster trust among the population in their health services. A healthier population is crucial for promoting economic progress and stability in Nigeria. Although Nigeria has progressed in specific areas of its healthcare system in the last decade, substantial deficiencies persist that necessitate collaborative efforts from all stakeholders, including government agencies, healthcare providers, and users, to establish a more responsive and effective healthcare framework in the future.

Supporting Information

S1 Checklist. Inclusivity in global research

S1 Checklist is a PLOS' policy on inclusivity in global research which focuses on ensuring that research conducted outside of a researcher's home country is reported transparently and ethically.

S1 Appendix. Table 1 Detailed Chi-Squared Crosstabulation

S1 Appendix provides a detailed representation of the crosstabulation of Sociodemographic attributes and average rating of the quality of the healthcare system.

S1 Appendix. Table 2 Detailed Chi-Squared Crosstabulation

S1 Appendix provides a detailed representation of the crosstabulation of sociodemographic attributes and preference of healthcare facilities.

References

1. Gyawali B, Khanal P, Mishra SR, van Teijlingen E, Wolf Meyrowitsch D. Building Strong Primary Health Care to Tackle the Growing Burden of Non-Communicable Diseases in Nepal. Glob Health Action. 2020;13:1788262.

2. Kirwan D. Global health: current issues, future trends and foreign policy. Clin Med. 2009;9:247–53.

3. US Institute of Medicine. The Changing Nature of Health Care [Internet]. National Academies Press (US); 2008 [cited 2022 Dec 3]. Available from: https://www.ncbi.nlm.nih.gov/books/NBK52825/

4. Ireland S. Revealed: Countries With The Best Health Care Systems, 2021 [Internet]. CEOWORLD Mag. 2021 [cited 2022 Dec 3]. Available from: https://ceoworld.biz/2021/04/27/revealed-countries-with-the-best-health-care-systems-2021/

5. WHO. Better non-communicable disease outcomes, challenges and opportunities for health systems, No. 2, Turkey Country Assessment. 2014;

6. Sachs J, Schmidt-Traub G, Kroll C, Lafortune G, Fuller G. Sustainable Development Report 2019 [Internet]. 2019 [cited 2023 Feb 11]. Available from: https://www.sustainabledevelopment.report

7. Ogaji D, Brisibe S. The Nigerian Health Care System: Evolution, contradictions, and proposal for future debates. P H Med J. 2015;9:79–88.

8. Etukumana EA, Orie JB. Health workers' perception on the work, working conditions, compensation, and carer development in a Nigerian tertiary hospital. Niger J Fam Pract. 2017;8:39–51.

9. Awosoga OA, Odunaiya NA, Oyewole OO, Ogunlana MO, Mbada CE, Onyeso OK, et al. Pattern and perception of wellbeing, quality of work life and quality of care of health professionals in Southwest Nigeria. BMC Health Serv Res. 2022;22:1387.

10. Lafiya. Options | Achieving health for all in Nigeria: what does it take? [Internet]. 2021 [cited 2023 Jan 14]. Available from: https://options.co.uk/news/achieving-health-for-all-in-nigeria-what-does-it-take

11. Welcome MO. The Nigerian health care system: Need for integrating adequate medical intelligence and surveillance systems. J Pharm Bioallied Sci. 2011;3:470–8.

12. Adeloye D, David RA, Olaogun AA, Auta A, Adesokan A, Gadanya M, et al. Health workforce and governance: the crisis in Nigeria. Hum Resour Health. 2017;15:32.

13. Exportiamo.it. Medical tourism, another drain on the economy [Internet]. Exportiamo.it. 2015 [cited 2023 Jan 14]. Available from: https://www.exportiamo.it/news/11680/medical-tourism-another-drain-on-the-economy/

14. IMTJ. Nigerian politicians kill medical tourism bill [Internet]. LaingBuisson News. 2019 [cited 2023 Jan 14]. Available from: https://www.laingbuissonnews.com/imtj/news-imtj/nigerian-politicians-kill-medical-tourism-bill/

15. WHO. UHC Roundtable [Internet]. 2018 [cited 2023 Jan 14]. Available from: https://www.who.int/director-general/speeches/detail/uhc-roundtable

16. Onwujekwe O, Ezumah N, Mbachu C, Obi F, Ichoku H, Uzochukwu B, et al. Exploring effectiveness of different health financing mechanisms in Nigeria; what needs to change and how can it happen? BMC Health Serv Res. 2019;19:661.

17. Akinleye DD, McNutt L-A, Lazariu V, McLaughlin CC. Correlation between hospital finances and quality and safety of patient care. PLOS ONE. 2019;14:e0219124.

18. Photius. The World Health Organization’s ranking of the world’s health systems, by Rank [Internet]. 2019 [cited 2023 Jan 14]. Available from: https://photius.com/rankings/healthranks.html

19. Statista. Health index of countries worldwide in 2021 [Internet]. Statista. 2022 [cited 2023 Jan 14]. Available from: https://www.statista.com/statistics/1290168/health-index-of-countries-worldwide-by-health-index-score/

20. WHO. World Health Organization Assesses the World’s Health Systems [Internet]. 2000 [cited 2023 Jan 14]. Available from: https://www.who.int/news/item/07-02-2000-world-health-organization-assesses-the-world's-health-systems

21. Nwanaji-Enwerem O, Bain P, Marks Z, Nwanaji-Enwerem P, Staton CA, Olufadeji A, et al. Patient satisfaction with the Nigerian National Health Insurance

Scheme two decades since establishment: A systematic review and recommendations for improvement. Afr J Prim Health Care Fam Med. 2022;14:3003.

22. USAID. Global Health | Nigeria | U.S. Agency for International Development [Internet]. 2017 [cited 2022 Dec 3]. Available from: https://www.usaid.gov/nigeria/global-health

23. UNICEF. Situation of women and children in Nigeria [Internet]. 2021 [cited 2023 Feb 11]. Available from: https://www.unicef.org/nigeria/situation-women-and-children-nigeria

24. The World Bank. Life expectancy at birth, total (years) - Nigeria | Data [Internet]. 2022 [cited 2022 Dec 3]. Available from: https://data.worldbank.org/indicator/SP.DYN.LE00.IN?locations=NG

25. Macrotrends. Nigeria Death Rate 1950-2022 [Internet]. 2022 [cited 2022 Dec 3]. Available from: https://www.macrotrends.net/countries/NGA/nigeria/death-rate

26. Edeh HC. Building Strong Primary Health Care to Tackle the Growing Burden of Non-Communicable Diseases in Nepal - PMC [Internet]. 2022 [cited 2023 Jan 14]. Available from: https://www.ncbi.nlm.nih.gov/pmc/articles/PMC7480568/

27. IHME. Nigeria [Internet]. Inst. Health Metr. Eval. 2015 [cited 2022 Dec 3]. Available from: https://www.healthdata.org/nigeria

28. Micah AE, Cogswell IE, Cunningham B, Ezoe S, Harle AC, Maddison ER, et al. Tracking development assistance for health and for COVID-19: a review of development assistance, government, out-of-pocket, and other private spending on health for 204 countries and territories, 1990–2050. The Lancet. 2021;398:1317–43.

29. Medicine I of M (US) R on E-B, Yong PL, Saunders RS, Olsen L. Transparency of Cost and Performance [Internet]. Healthc. Imperative Low. Costs Improv. Outcomes Workshop Ser. Summ. National Academies Press (US); 2010 [cited 2023 Jan 14]. Available from: https://www.ncbi.nlm.nih.gov/books/NBK53921/

30. Aregbeshola BS, Folayan MO. Nigeria's financing of health care during the COVID-19 pandemic: Challenges and recommendations. World Med Health Policy. 2022;14:195–204.

31. ITA. Nigeria - Healthcare [Internet]. 2021 [cited 2022 Dec 3]. Available from: https://www.trade.gov/country-commercial-guides/nigeria-healthcare

32. Onwuzoo A. Nigeria’s healthcare system in pitiable state, bleeding –Physician [Internet]. Healthwise. 2021 [cited 2023 Jan 14]. Available from: https://healthwise.punchng.com/nigerias-healthcare-system-in-pitiable-state-bleeding-physician/

33. Tulchinsky TH, Varavikova EA. Chapter 13 - National Health Systems. In: Tulchinsky TH, Varavikova EA, editors. New Public Health Third Ed [Internet]. San Diego: Academic Press; 2014 [cited 2022 Dec 3]. p. 643–728. Available from: https://www.sciencedirect.com/science/article/pii/B9780124157668000136

34. Cohen J. China Protests Go Beyond Demanding An End To Severe Covid-19 Restrictions [Internet]. Forbes. 2022 [cited 2023 Feb 11]. Available from: https://www.forbes.com/sites/joshuacohen/2022/12/01/china-protests-go-beyond-demanding-an-end-to-severe-covid-19-restrictions/

35. Worldometer. Nigeria Population (2024) - Worldometer [Internet]. Niger. Popul. 2024. 2024 [cited 2024 Feb 2]. Available from: https://www.worldometers.info/world-population/nigeria-population/

36. Olu-Adeyemi L. Federalism in Nigeria -Problems, Prospects and the Imperative of Restructuring. 2017;

37. RAND Corporation. Health Surveys [Internet]. 2023 [cited 2023 Jan 14]. Available from: https://www.rand.org/health-care/surveys_tools.html

38. SpinWheelApp. Editor | Spin the Wheel - Random Picker [Internet]. Wheel Mak. 2023 [cited 2023 Dec 17]. Available from: https://spinthewheel.app/0bhxMPPkoe

39. WMA. WMA - The World Medical Association-WMA Declaration of Helsinki – Ethical Principles for Medical Research Involving Human Subjects [Internet]. 2022 [cited 2023 Feb 11]. Available from: https://www.wma.net/policies-post/wma-declaration-of-helsinki-ethical-principles-for-medical-research-involving-human-subjects/

40. Budgetoffice. 2022 Budget - Budget Office of the Federation - Federal Republic of Nigeria [Internet]. 2023 [cited 2023 Dec 4]. Available from: https://www.budgetoffice.gov.ng/index.php/resources/internal-resources/budget-documents/2022-budget

41. WHO. Nigeria: health expenditure as share of GDP [Internet]. Statista. 2022 [cited 2024 Oct 5]. Available from: https://www.statista.com/statistics/1126455/health-expenditure-as-share-of-gdp-in-nigeria/

42. OECD. Health expenditure and financing [Internet]. 2023 [cited 2023 Nov 25]. Available from: https://stats.oecd.org/index.aspx?DataSetCode=SHA

43. Balogun JA. A Qualitative Investigation of the Barriers to the Delivery of High-Quality Healthcare Services in Nigeria. In: Balogun JA, editor. Niger Healthc Syst Pathw Univers High-Qual Health Care [Internet]. Cham: Springer International Publishing; 2021 [cited 2024 Feb 3]. p. 345–59. Available from: https://doi.org/10.1007/978-3-030-88863-3_11

44. Onwujekwe O, Ezumah N, Mbachu C, Obi F, Ichoku H, Uzochukwu B, et al. Exploring effectiveness of different health financing mechanisms in Nigeria; what needs to change and how can it happen? BMC Health Serv Res. 2019;19:661.

45. Adewole DA, Adeniji FIP, Adegbrioye SE, Dania OM, Ilori T. Enrollees' Knowledge and Satisfaction with National Health Insurance Scheme Service Delivery in a Tertiary Hospital, South West Nigeria. Niger Med J J Niger Med Assoc. 2020;61:27–31.

46. Akande RO, Abodunrin OL, Olarewaju SO, Adeomi AA, Akande JO, Faramade IO. Health insurance in private and public health facilities in Southwestern Nigeria: what determines clients´ satisfactionwith quality of service? Pan Afr Med J [Internet]. 2022 [cited 2023 Sep 21];41. Available from: https://www.panafrican-med-journal.com/content/article/41/268/full

47. Akintobi TH, Lockamy E, Goodin L, Hernandez ND, Slocumb T, Blumenthal D, et al. Processes and Outcomes of a Community-Based Participatory Research-Driven Health Needs Assessment: A Tool for Moving Health Disparity Reporting to Evidence-Based Action. Prog Community Health Partnersh Res Educ Action. 2018;12:139–47.

48. Akinyinka MR, Oluwole EO, Odusanya OO. Community perception of quality of health care received and client satisfaction in Lagos, Nigeria. J Community Med Prim Health Care. 2019;31:47–65.

49. Akinyinka MR, Oluwole EO, Odusanya OO. Predictors of Client Satisfaction Among Recent Users of Health Services in Lagos, Nigeria. Health Serv Insights. 2020;13:1178632920934499.

50. ITA. Nigeria - Healthcare [Internet]. 2021 [cited 2022 Dec 4]. Available from: https://www.trade.gov/country-commercial-guides/nigeria-healthcare

51. Yong PL, Olsen L. Transparency of Cost and Performance [Internet]. National Academies Press (US); 2010 [cited 2023 Jan 14]. Available from: https://www.ncbi.nlm.nih.gov/books/NBK53921/

52. Ireland S. Revealed: Countries With The Best Health Care Systems, 2021 [Internet]. CEOWORLD Mag. 2021 [cited 2022 Dec 4]. Available from: https://ceoworld.biz/2021/04/27/revealed-countries-with-the-best-health-care-systems-2021/

53. Tulchinsky TH, Varavikova EA. Chapter 13 - National Health Systems. In: Tulchinsky TH, Varavikova EA, editors. New Public Health Third Ed [Internet]. San Diego: Academic Press; 2014 [cited 2022 Dec 3]. p. 643–728. Available from: https://www.sciencedirect.com/science/article/pii/B9780124157668000136

Fig 1 Click here to access/download;Figure;Fig1.tif

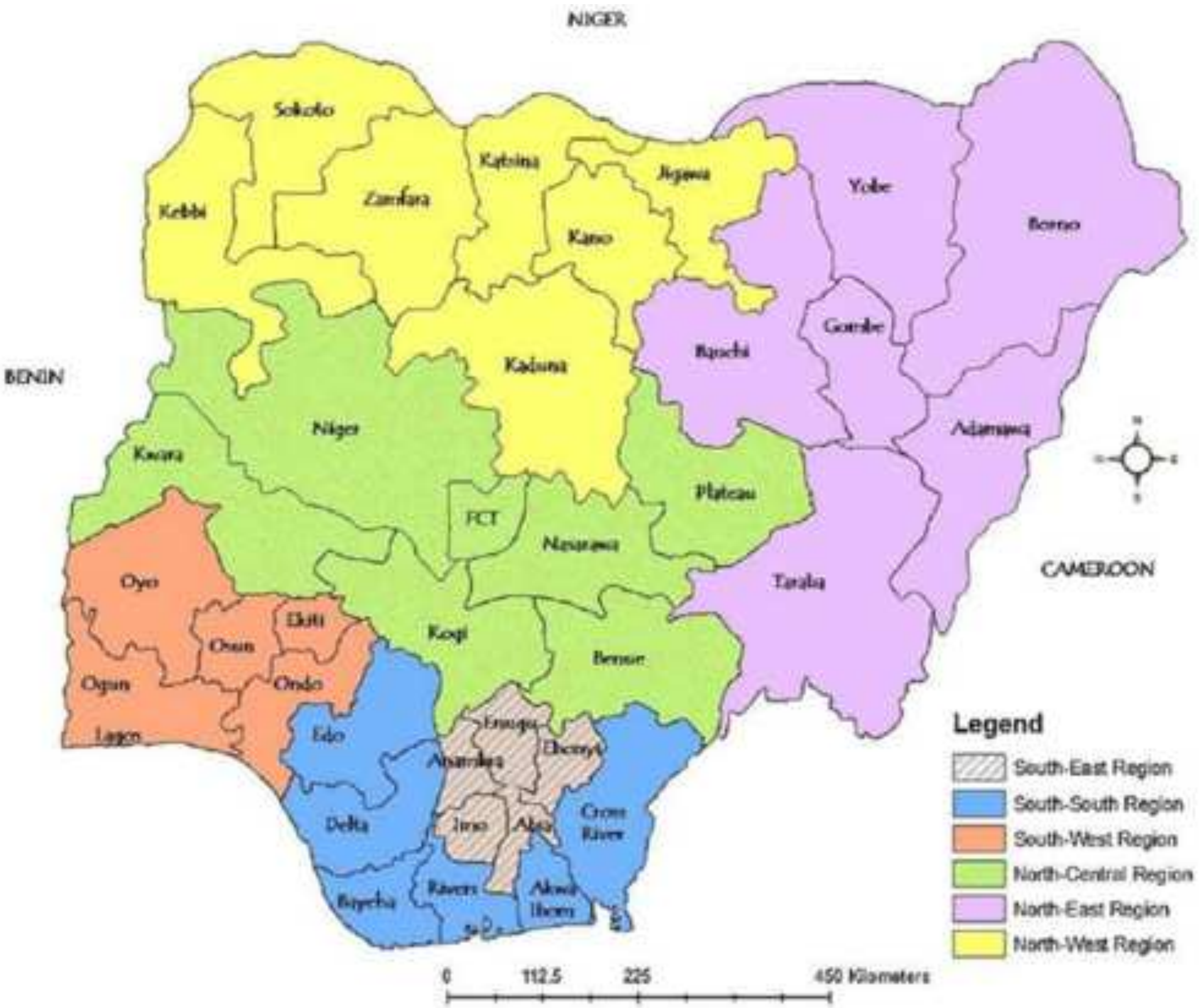

Fig 2

Click here to access/download;Figure;Fig2.tif

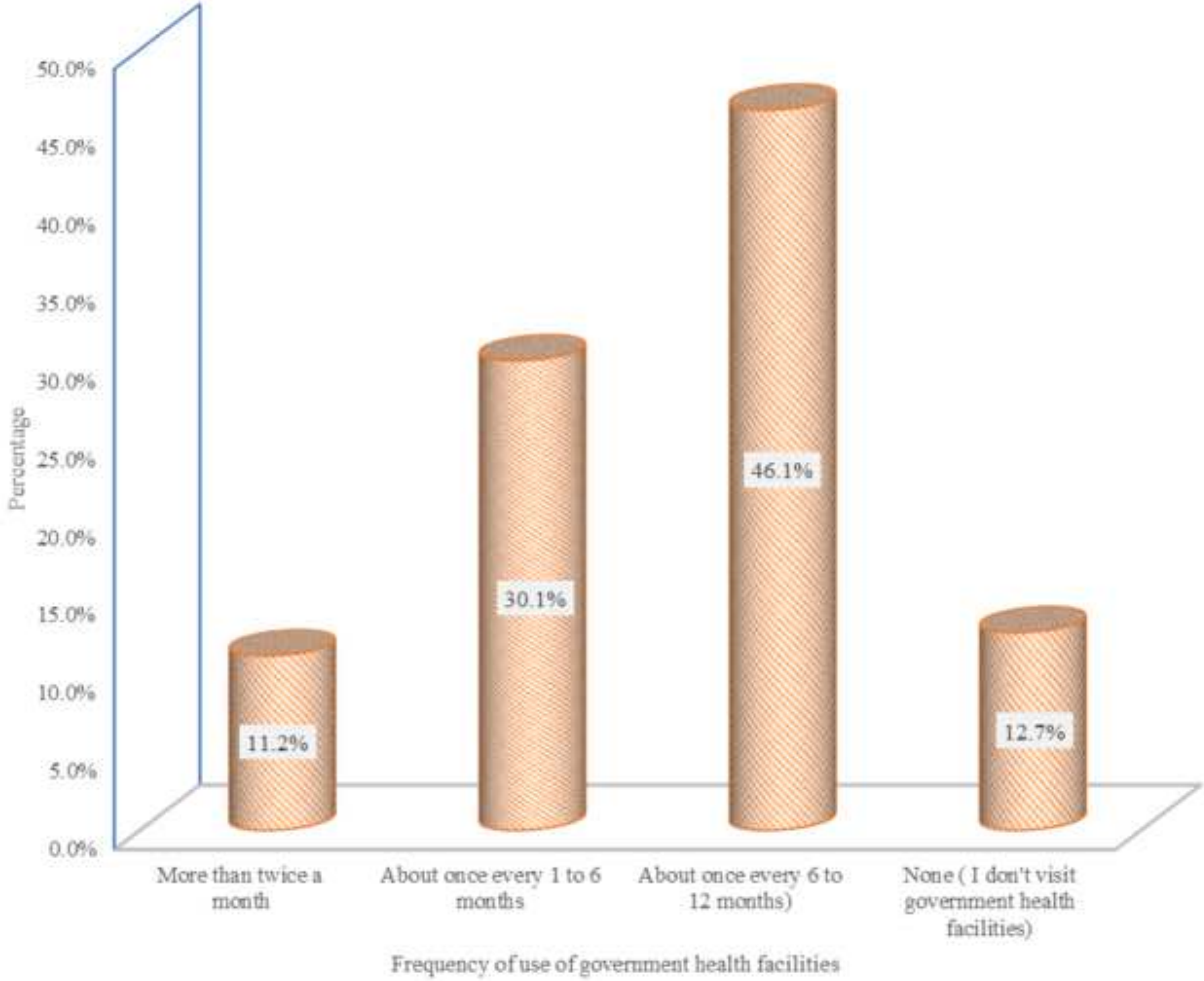

Fig 3

Click here to access/download;Figure;Fig3.tif

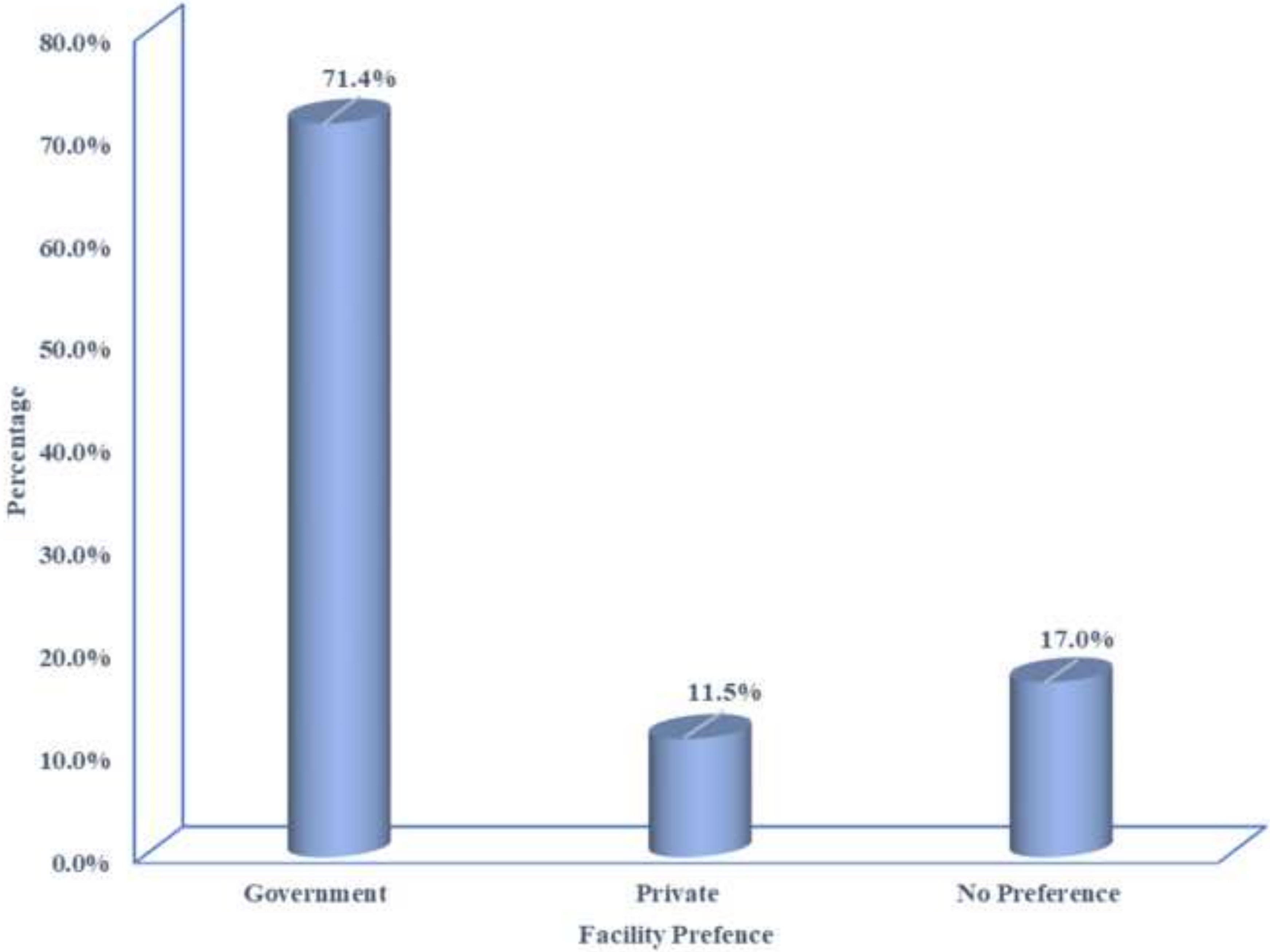

Fig 4

Click here to access/download;Figure;Fig4.tif

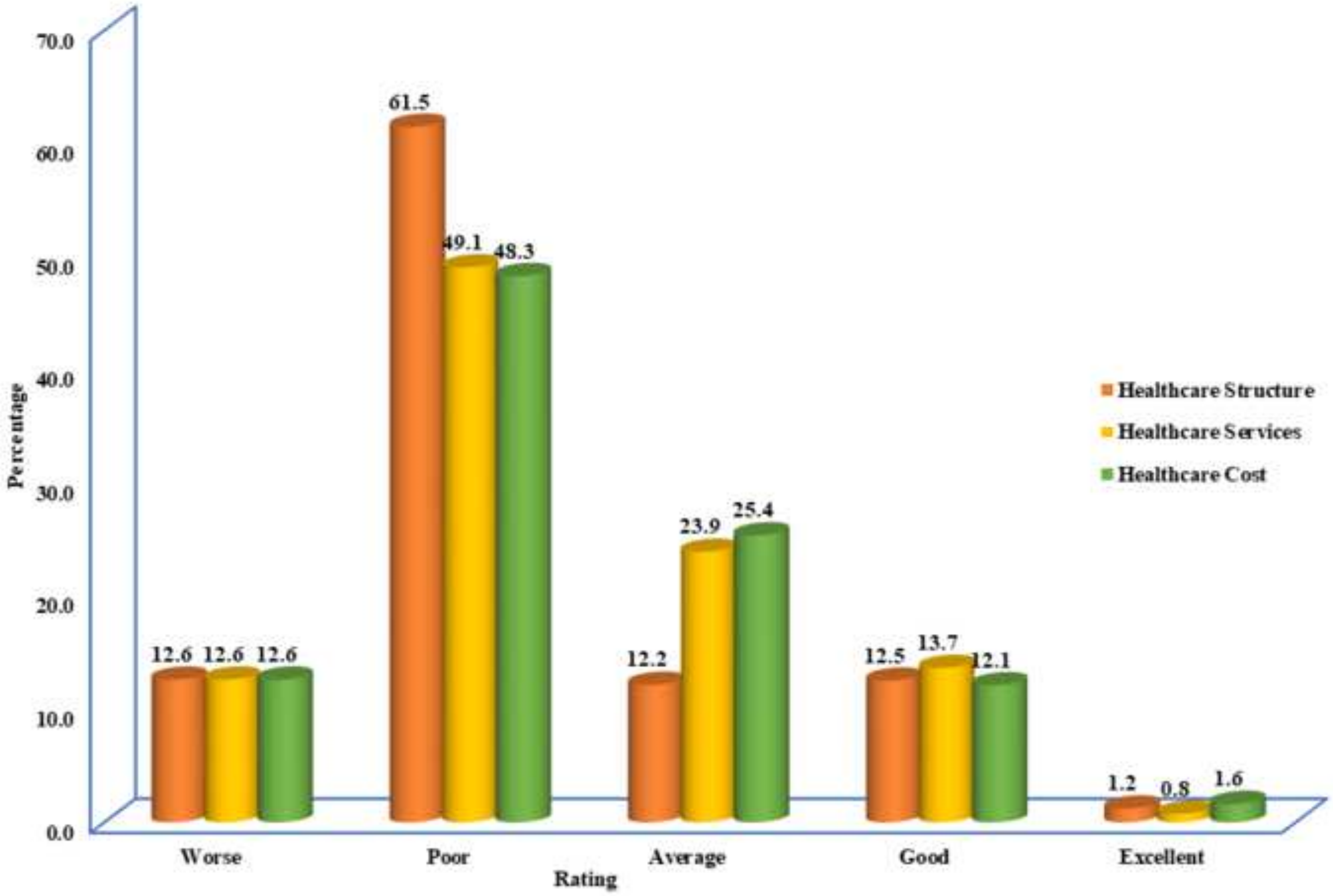

Fig 5

Click here to access/download;Figure;Fig5.tif

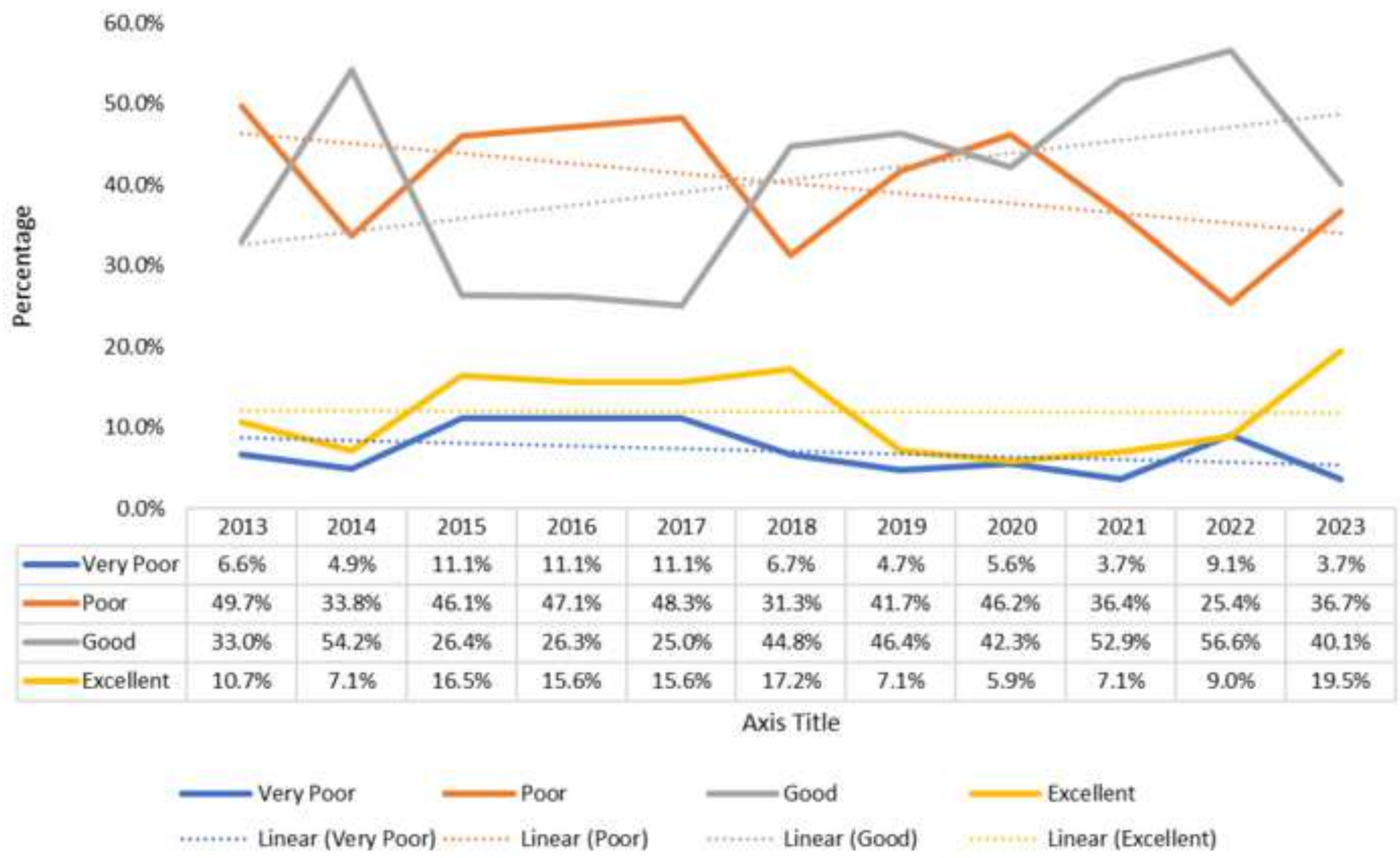

	2013	2014	2015	2016	2017	2018	2019	2020	2021	2022	2023
Very Poor	6.6%	4.9%	11.1%	11.1%	11.1%	6.7%	4.7%	5.6%	3.7%	9.1%	3.7%
Poor	49.7%	33.8%	46.1%	47.1%	48.3%	31.3%	41.7%	46.2%	36.4%	25.4%	36.7%
Good	33.0%	54.2%	26.4%	26.3%	25.0%	44.8%	46.4%	42.3%	52.9%	56.6%	40.1%
Excellent	10.7%	7.1%	16.5%	15.6%	15.6%	17.2%	7.1%	5.9%	7.1%	9.0%	19.5%

Fig 6

Click here to access/download;Figure;Fig6.tif

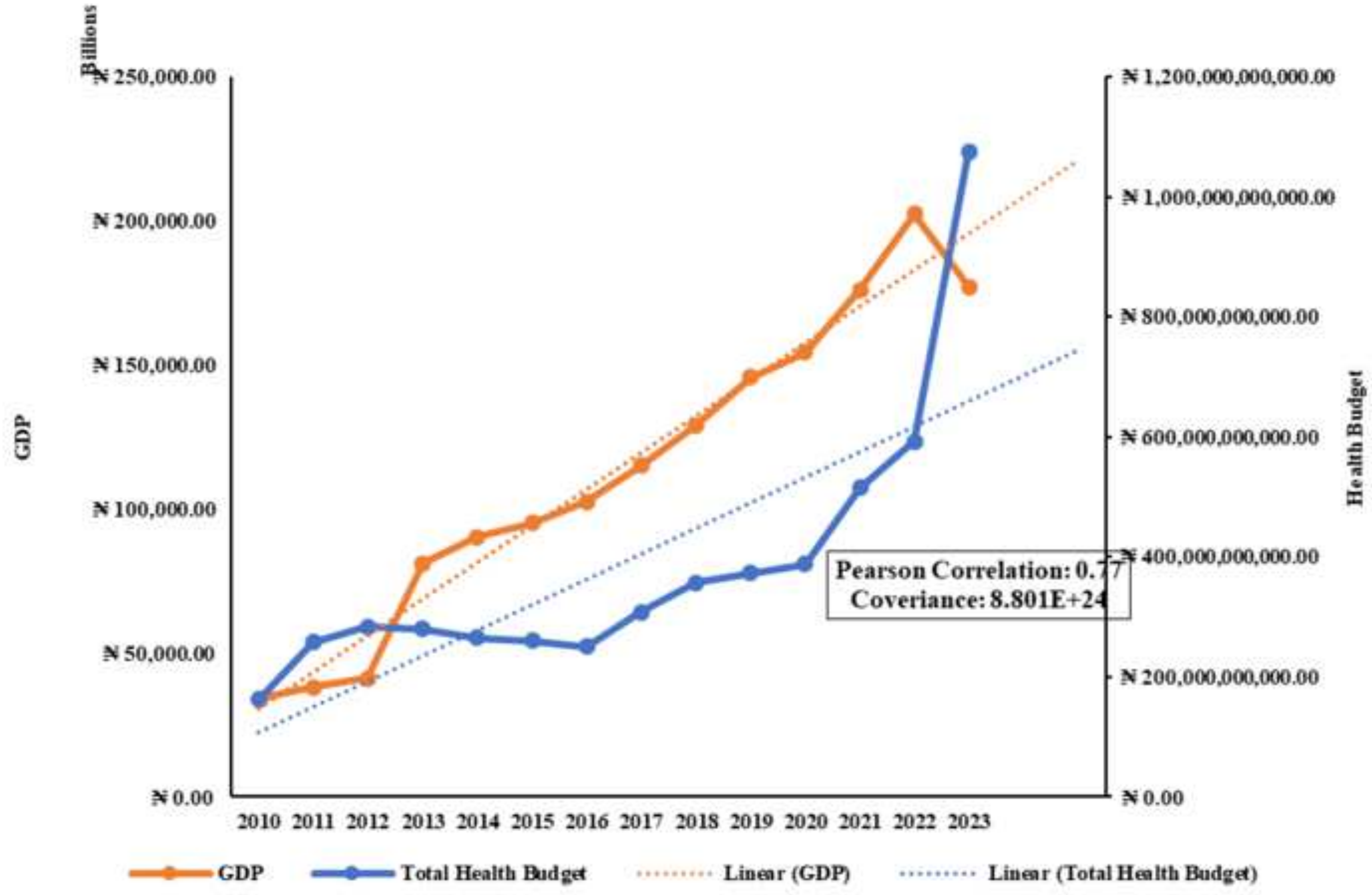

Fig 7

Click here to access/download;Figure;Fig7.tif

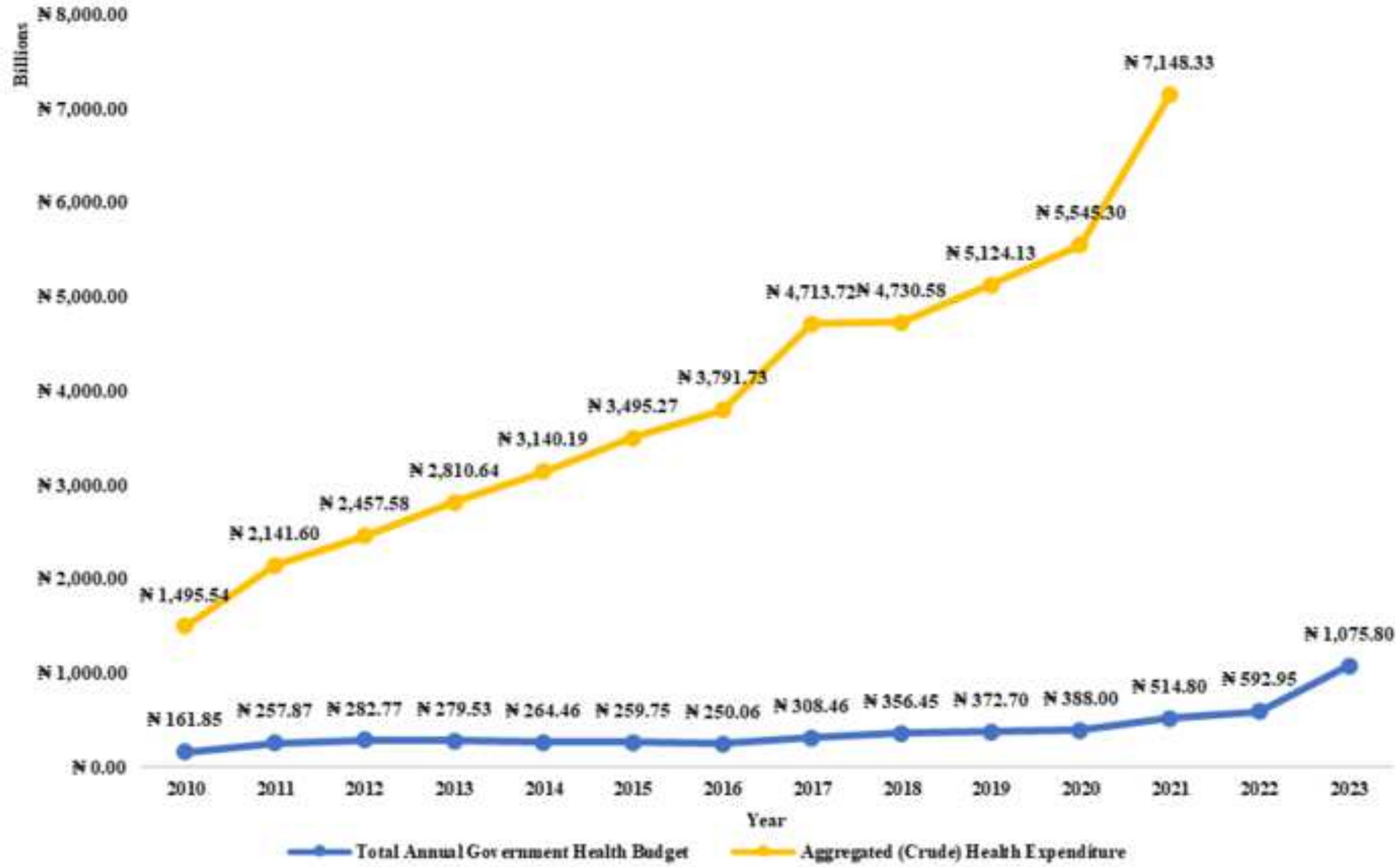

Fig 8

Click here to access/download;Figure;Fig8.tif

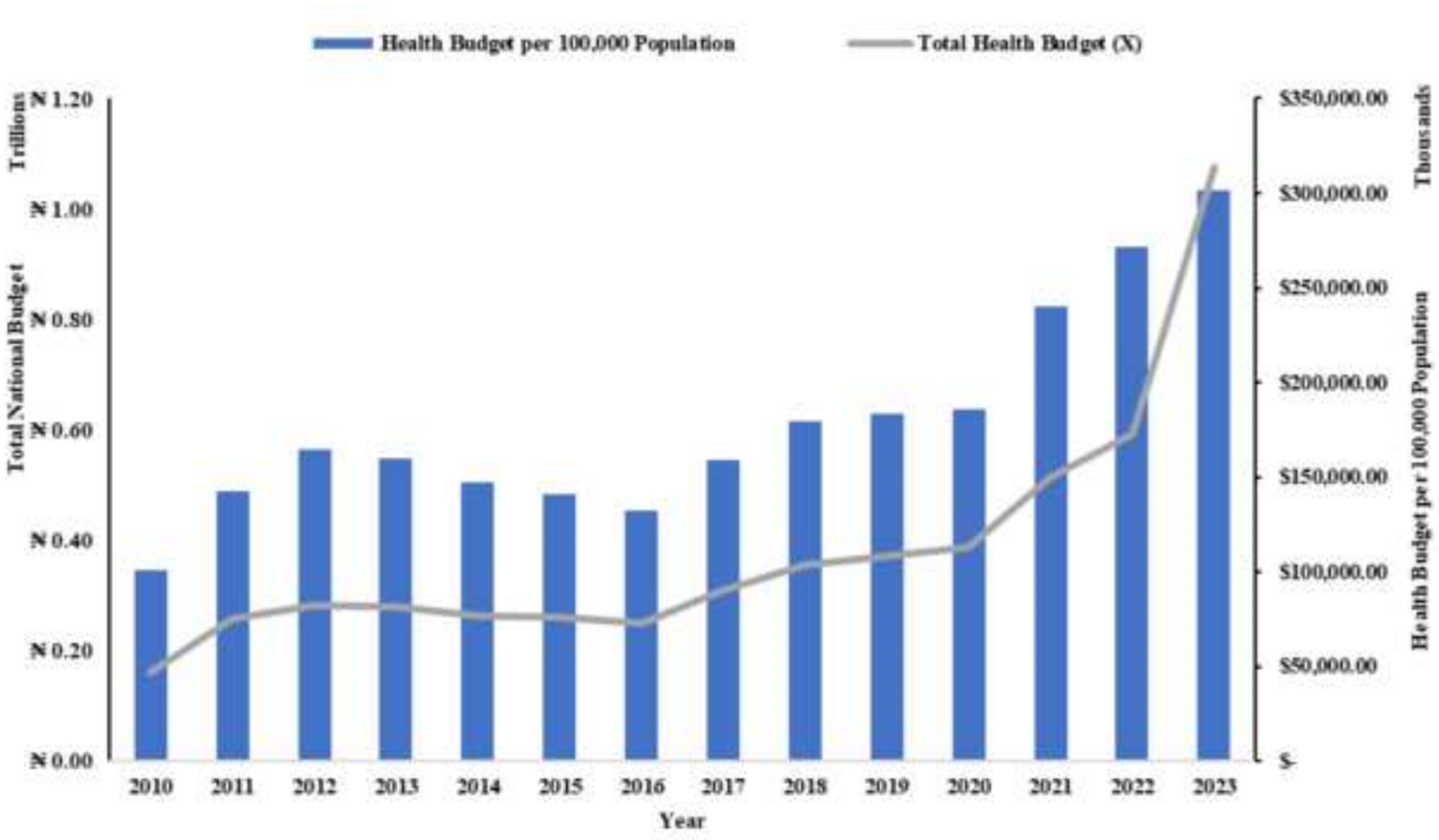

Fig 9

Click here to access/download;Figure;Fig9.tif

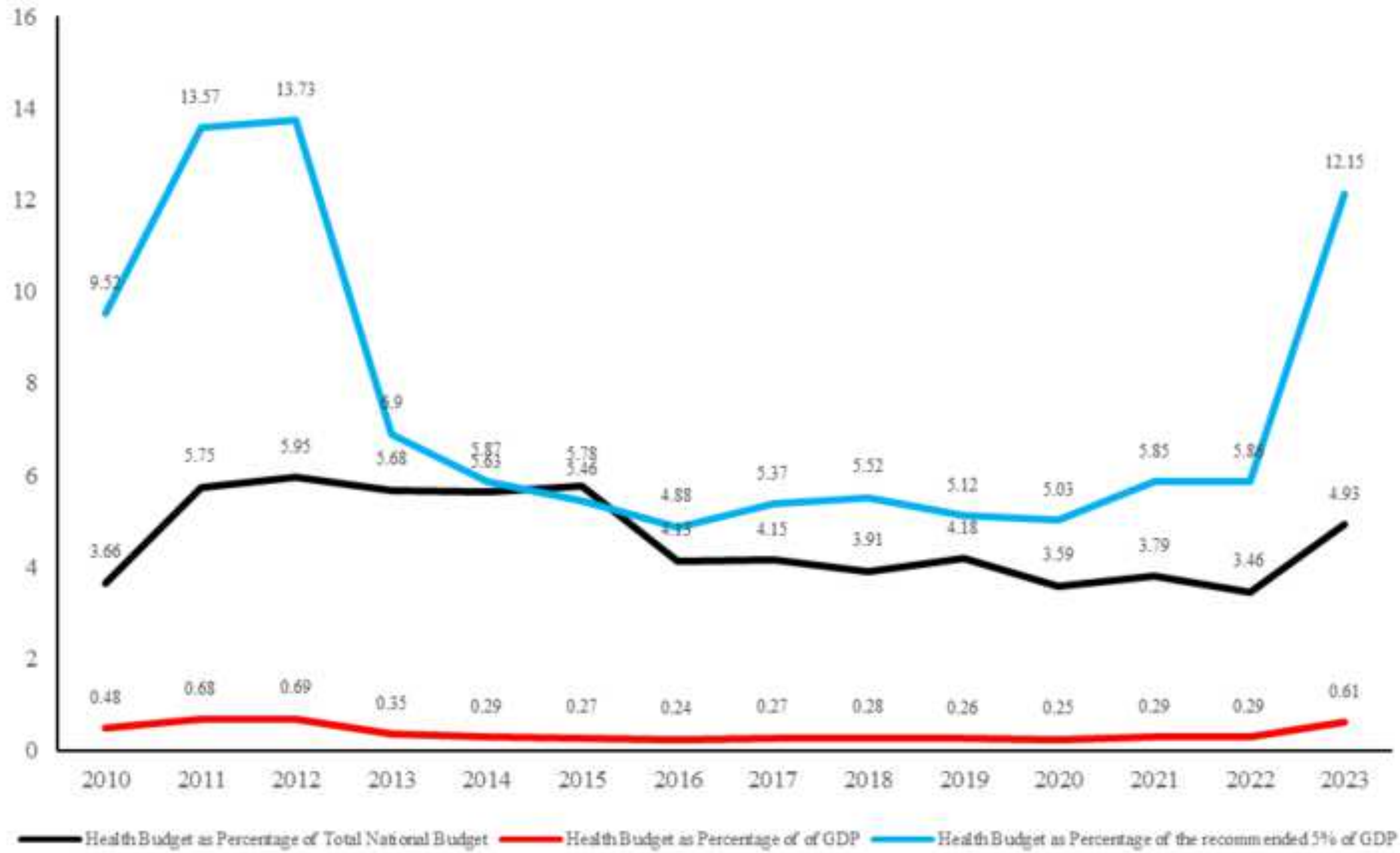

Fig 10

Click here to access/download;Figure;Fig10.tif

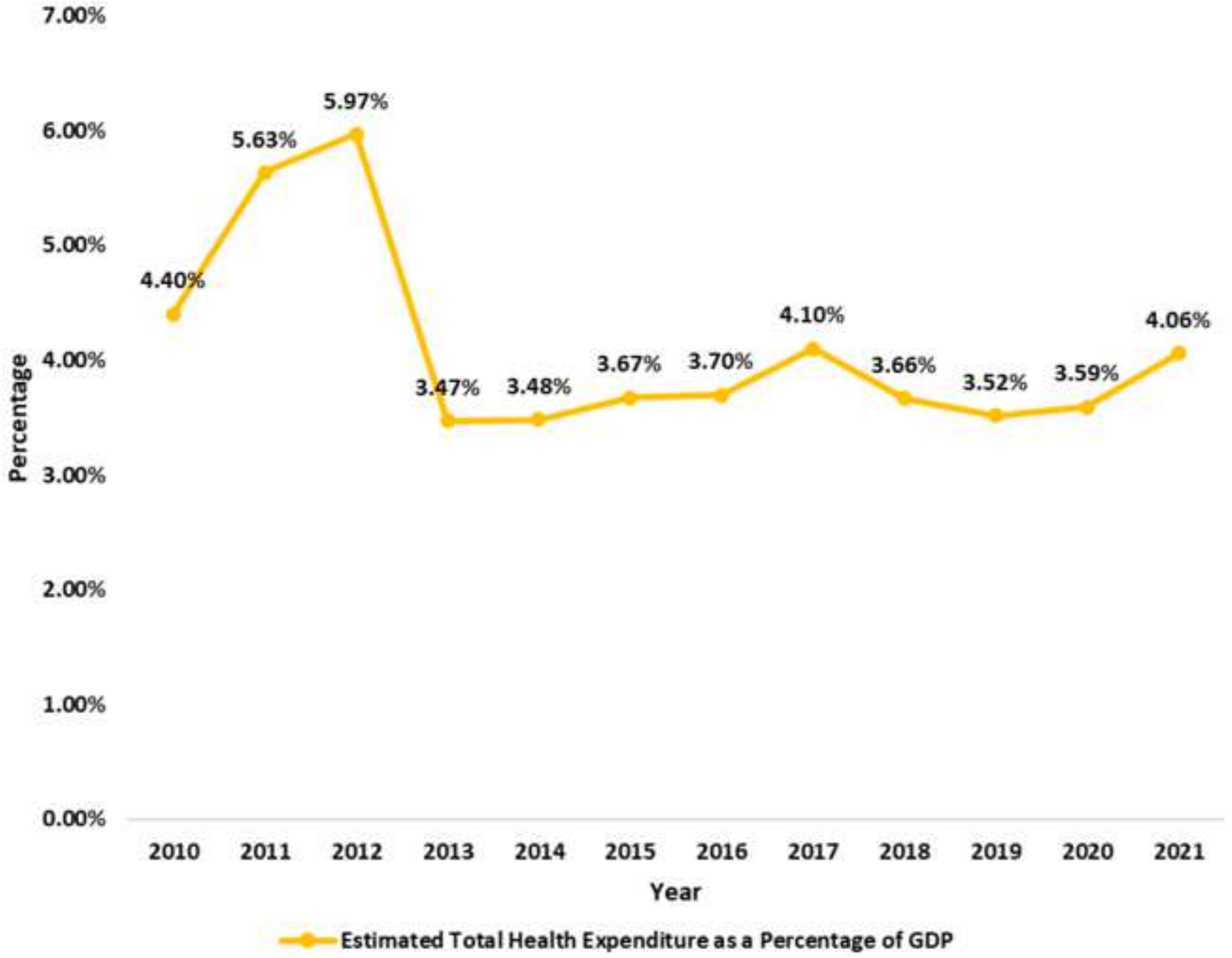

Fig 11

Click here to access/download;Figure;Fig11.tif

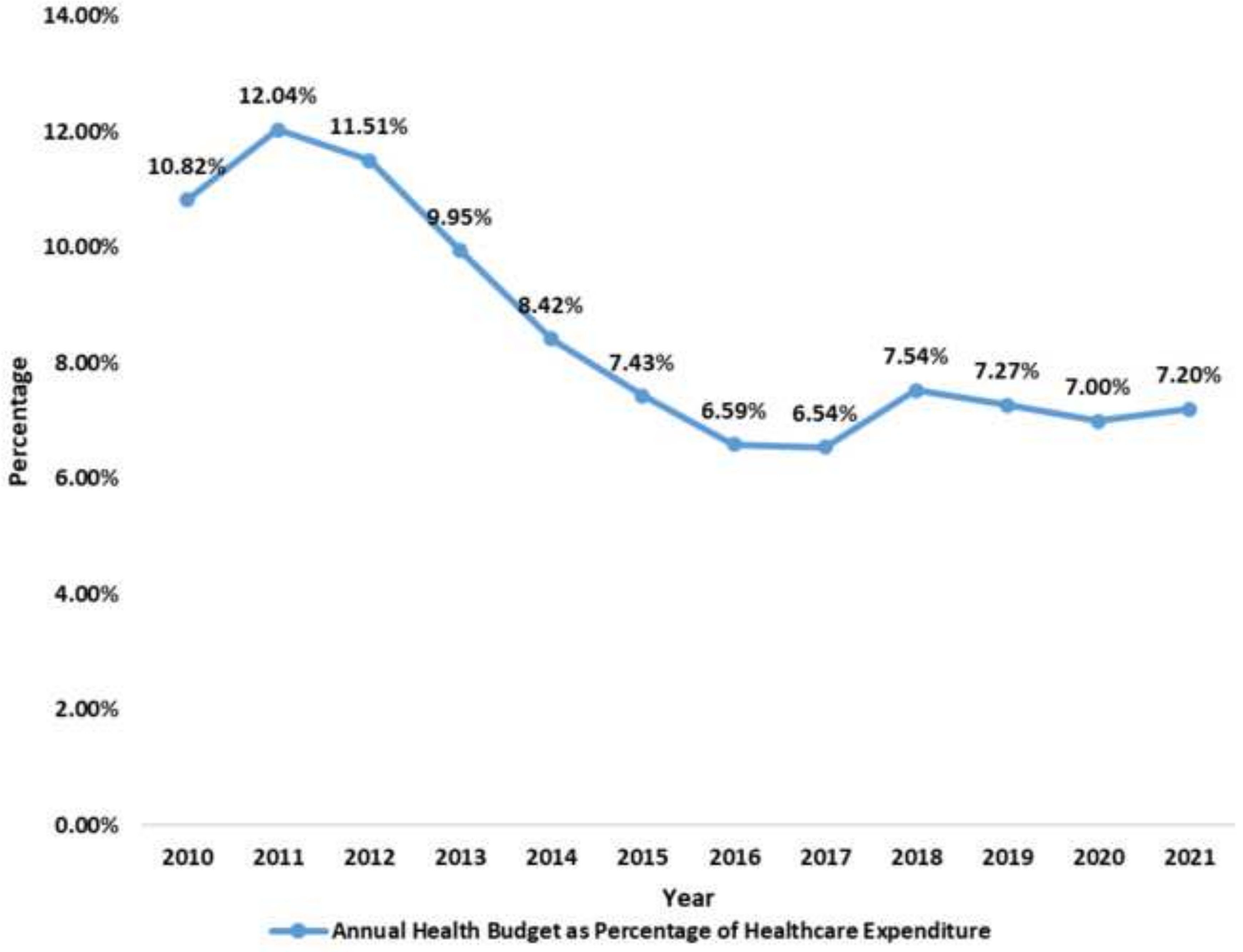

S1 Inclusivity in global research Checklist

Click here to access/download
Supporting Information
S1_Inclusivity_in_global_research_Checklist.docx

S1 Table

Click here to access/download
Supporting Information
S1_Table.docx

S2 Table

Click here to access/download
Supporting Information
S2_Table.docx
