# Supplementary material for: Critical review of healthcare financing and a survey of system quality perception among healthcare users in Nigeria (2010–2023)
Source: PLOS Glob Public Health. 2025 May 28;5(5):e0004615. doi: 10.1371/journal.pgph.0004615 (PMC12118842; doi:10.1371/journal.pgph.0004615)
Supplement: Attachment — Submitted filename: Reponses to Reviewers.docx [file pgph.0004615.s006.docx]

**Responses to Reviewer’s Comments**

Dear Reviewers,

Thank you for dedicating your time and effort to thoroughly reviewing our manuscript. We greatly appreciate your detailed analysis and constructive, encouraging feedback. You have highlighted a few concerns regarding specific aspects of the manuscript. We have carefully addressed these issues and implemented the necessary corrections.

The attached PDF includes two documents with tracked changes. The first highlights the main corrections, while the second focuses on minor adjustments to grammar and punctuation. At the top, you'll find the final version, incorporating all the revisions described below. We apologize for the document's length, which was necessary due to the asynchronous updates made by different authors.

B.O. Josiah,

*Corresponding*

**1. Verification of Data and Rankings:**

The manuscript states that Nigeria ranks 74th out of 89 countries and is 159th out of 162 on the SDG index. Clarifying the context behind the “89 countries” which was a standalone statistic in the abstract would be helpful.

**2. Updating Index Data:**

If available, please cite the most recent statistics for the background. For example, the updated SDG report by Sachs et al (2024) ranks Nigeria 146th of 166 countries, rather than the previous 159th position out of 162 countries in 2019.

Incorporating updated data can help strengthen the policy implications of this important study.

**Response**: *We reviewed this data and updated it to the latest statistics as recommended. The new text now reads* “Nigeria aims to enhance its healthcare quality index score of 84th out of 110 countries and its Sustainable Development Goals Index ranking of 146th out of 166.” *The initial ranking (84/110) is based on the CEOWORLD Magazine Health Care Index, which uses traditional and patient-based reviews to rank healthcare systems. The entries have been corrected throughout the document.*

**3.Healthcare Expenditure/Secondary Data**

Please, verify the claim that Nigeria allocates only 0.37% of GDP to healthcare. Provide the source and context (e.g., whether this was based on government data, a questionnaire, or budget analysis from the nine selected states).

Please, clearly explain the methodology used to obtain the budget data before the analyses, clarifying the specific reputable government and online sources in Refs 40 - 42 and whether all the data are publicly available, or were privately sourced.

The transparency of the budget data claims in this study is especially important to support this interesting argument in the discussion: "This contrasts with many published and often misleading claims that Nigerian government budget is as high as 4% to 7% of national budget for healthcare".

**Response**: *We have amended the methodology to clearly reflect the data sources used in the study. All budget data were sourced from the official budget repository (Budgetoffice) with the guidance of the Federal Ministry of Health, Ministry of Finance, and the Ministry of Budget and Economic Planning Abuja.*

*Data related to expenditure was calculated using the WHO’s Global Health Expenditure Database; WHO’s Nigeria: health expenditure as share of GDP, and the WHO’s Current health expenditure (CHE) as percentage of gross domestic product (GDP) (%) - Data by country. We have updated the methods with this information and ensured that every other data used is also cited accordingly.*

**4. Additional Clarifications and Methodological Details**

In reporting the sample size calculation, please include an appropriate citation (e.g Cochran, 1977).

Re-examine statements such as “Using (the)Lagos, with the highest adult population”, and a couple more across the manuscript for clarity and proper punctuation.

**Response***: We have amended this statement to reflect the specific name “Cochran formula” and added the appropriate citation. The new text now reads* “The sample size for each state was calculated using Lagos, which has the largest adult population, as the reference population proportion. The sample for Lagos was initially determined using the Cochrane formula (Z^2^*p*q / e^2^) [38]; where Z = 1.96 at 95% confidence interval, p = 0.5 (incidence rate), q = 0.5 (non–incidence rate) and e = 0.05 (margin of error). The calculation yielded a sample of 384, which was approximated to 390. The population-to-sample ratio derived from Lagos was then applied to other states, resulting in a target total sample size of 1,830 (Table 1).”

**5. Punctuation and Text Formatting Consistency:**

Please, carefully address the typing errors and formatting inconsistencies throughout the manuscript. For example, check the sentence: “The survey was conducted between 6/8/2023 and 8/18/2023 and the data was collated with Google…” and ensure that dates, figures, and numerical data (e.g., population numbers, financial figures) are consistently formatted, and et al is italicized.

**Response**: We reviewed the document multiple times to ensure consistency in text formatting and punctuation based on all the recommendations.

6. Affiliations and Institutional Details:

Confirm that all author affiliations are complete and accurate. For instance, verify that entries such as “Irrua Specialist Teaching Hospital” include the necessary details (state, country, etc.).

**Response**: We have reviewed the affiliation details and added the state and country to those identified by you, as well as the others with similar missing information.

Reviewer #2: I eulogize the authors for the good work done. It was a very good study which highlighted the challenges confronting the healthcare delivery system in Nigeria. I observed some minor issues in the manuscript that the authors need to address.

Also, some sentences and paragraphs were repeated which should be deleted.

I also observed that some percentages were written to two decimal places while all the others were to just one decimal space, the authors should look into that as well.

By and large, it was a well-written manuscript.

**Response**: Thank you again for the powerful feedback. We have removed all repeated entries because of cloud-based tracked collaboration on word processor brands and versions across different operating systems (Windows, Mac, etc.) used by contributors.

The percentages have been harmonised to two decimal points throughout the manuscript, except for general data, such as the 5% of GDP recommendation on healthcare and the Abuja declaration of 15%.

6. PLOS authors have the option to publish the peer review history of their article ([what does this mean?](https://track.editorialmanager.com/CL0/https:%2F%2Fjournals.plos.org%2Fglobalpublichealth%2Fs%2Feditorial-and-peer-review-process%23loc-peer-review-history/1/010f0195ad02be94-ea2356dd-ab90-4712-a966-251eb17a9c1a-000000/4dytgLyv467_3OrC1p1cniVZblYA51TgXUiykZd8OYk=203)). If published, this will include your full peer review and any attached files.

**Do you want your identity to be public for this peer review?** If you choose “no”, your identity will remain anonymous but your review may still be made public.

For information about this choice, including consent withdrawal, please see our [Privacy Policy](https://track.editorialmanager.com/CL0/https:%2F%2Fwww.plos.org%2Fprivacy-policy/1/010f0195ad02be94-ea2356dd-ab90-4712-a966-251eb17a9c1a-000000/kNCcH4pMg_WDNu087vxZKQSExfkgOvaFj-ZExB_aHD8=203).

Reviewer #1: No

Reviewer #2: **Yes:** Ezra Olatunde Ogundare
